# A space for learning: An analysis of research on active learning spaces

**DOI:** 10.1016/j.heliyon.2019.e02967

**Published:** 2019-12-24

**Authors:** Robert Talbert, Anat Mor-Avi

**Affiliations:** a Department of Mathematics, Grand Valley State University, 1 Campus Drive, Allendale, 49401, MI USA; b College of Architecture, Illinois Institute of Technology, 3360 S. State St., Chicago, 60616, IL USA

**Keywords:** Education, Institutional culture, Active learning space, Active learning, 21st century classroom, Active learning classroom, Collaborative learning

## Abstract

Active Learning Classrooms (ALCs) are learning spaces specially designed to optimize the practice of active learning and amplify its positive effects in learners from young children through university-level learners. As interest in and adoption of ALCs has increased rapidly over the last decade, the need for grounded research in their effects on learners and schools has grown proportionately. In this paper, we review the peer-reviewed published research on ALCs, dating back to the introduction of “studio” classrooms and the SCALE-UP program up to the present day. We investigate the literature and summarize findings on the effects of ALCs on learning outcomes, student engagement, and the behaviors and practices of instructors as well as the specific elements of ALC design that seem to contribute the most to these effects. We also look at the emerging cultural impact of ALCs on institutions of learning, and we examine the drawbacks of the published research as well as avenues for potential future research in this area.

## 1 Introduction

### 1.1 What is active learning, and what is an active learning classroom?

**Figure 1:**
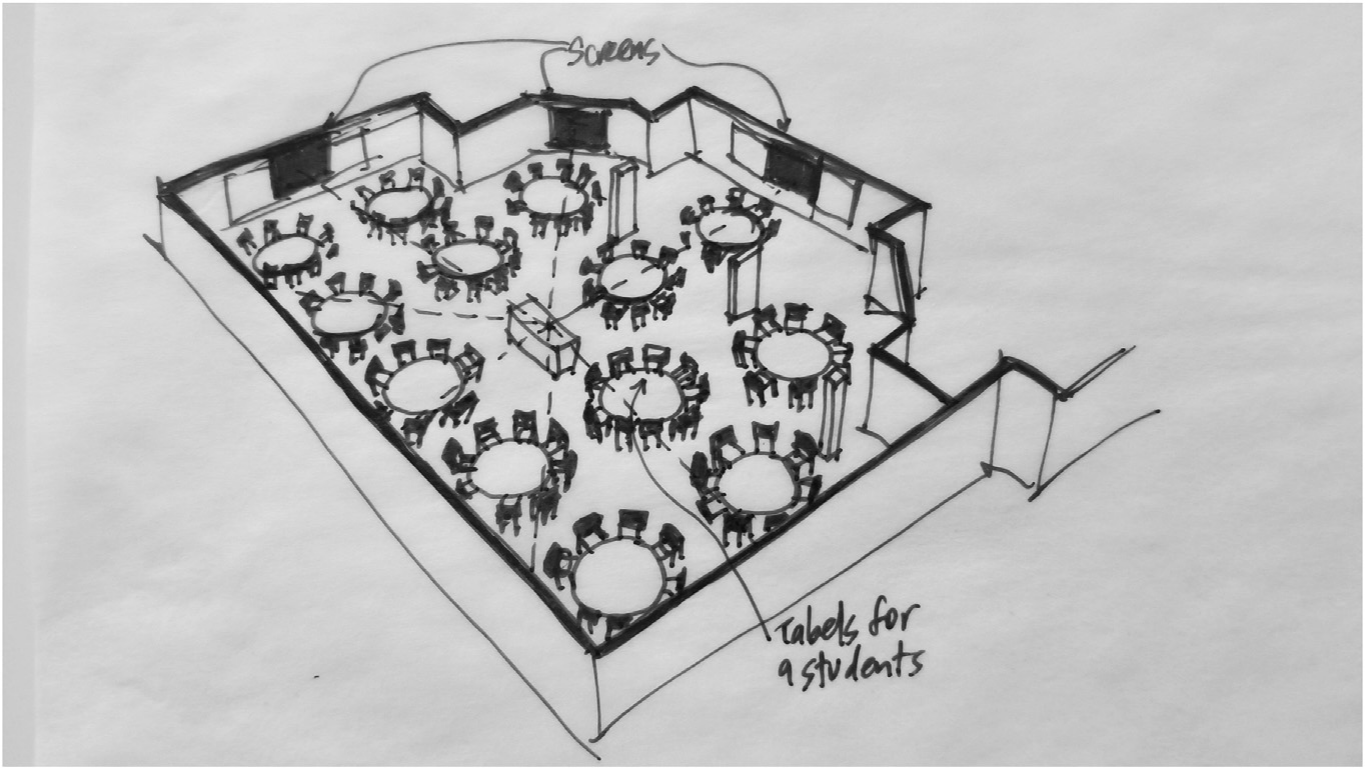
SCALE-UP classroom, adapted from http://scaleup.ncsu.edu/FAQs.html.

**Figure 2:**
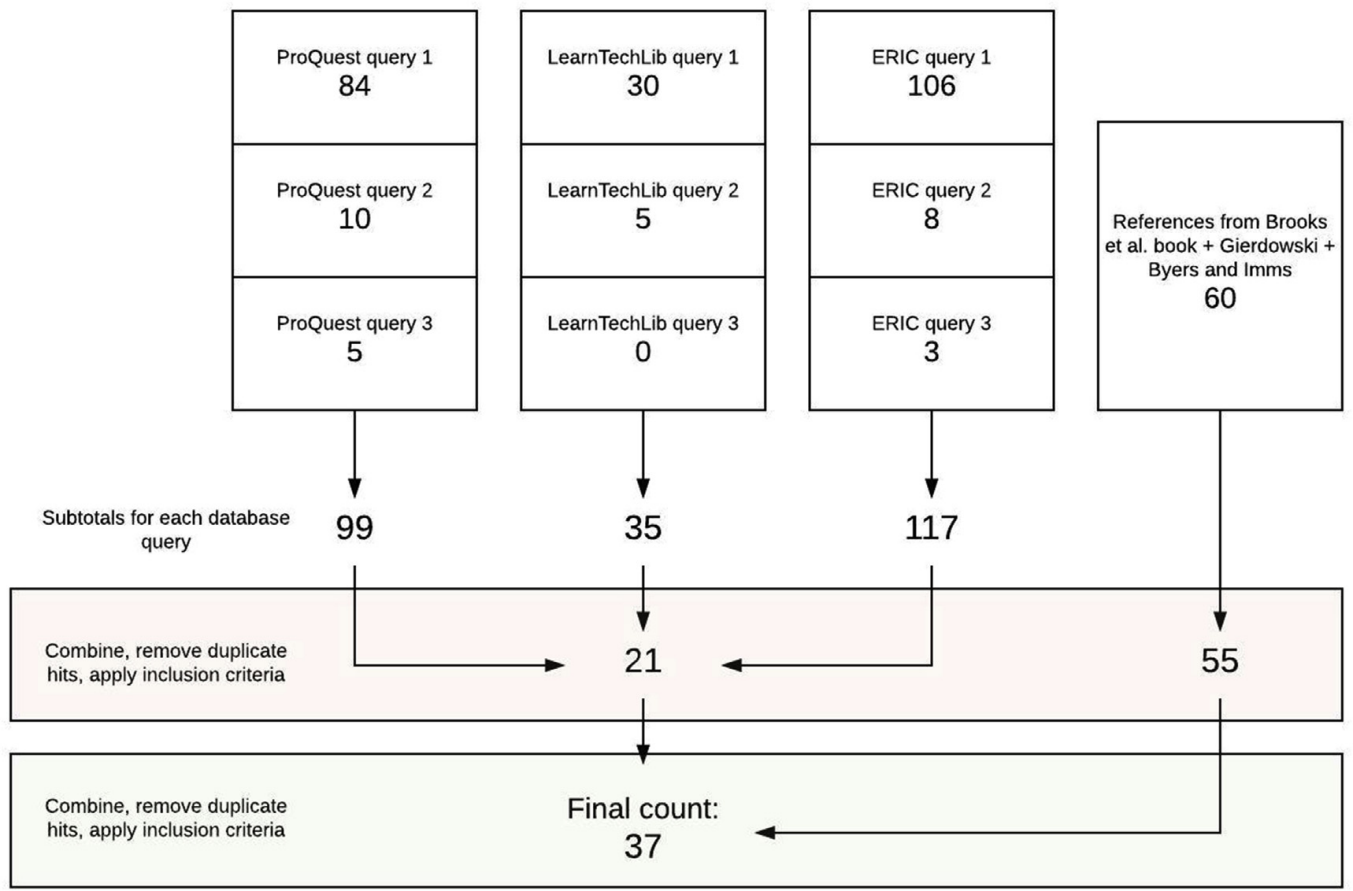
Database search overview.

**Figure 3:**
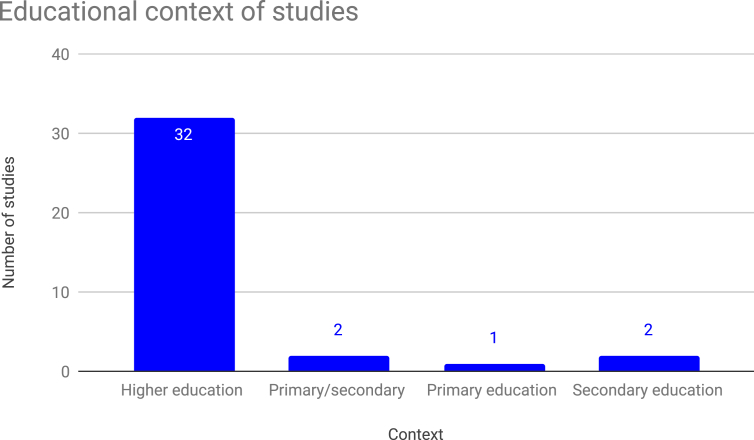
Number of studies by educational context.

**Figure 4:**
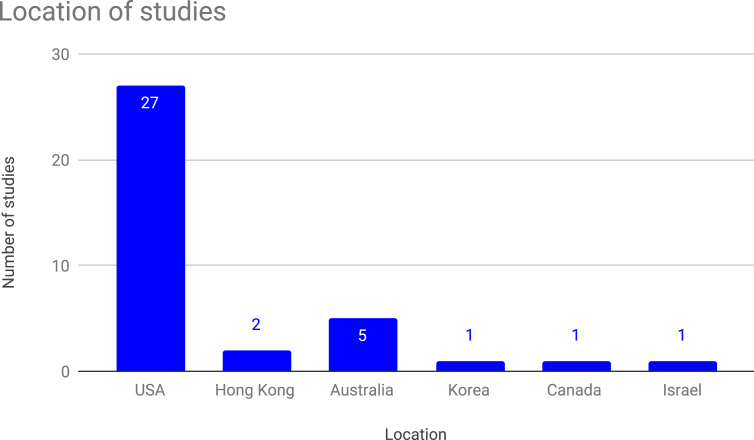
Number of studies by location.

**Figure 5:**
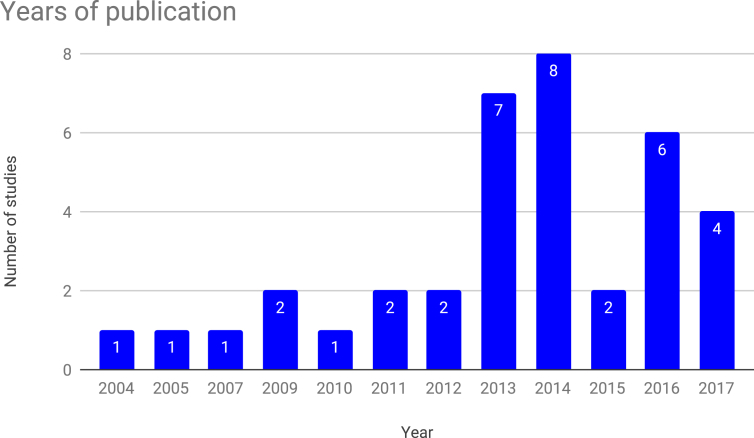
Number of studies by publication year.

**Figure 6:**
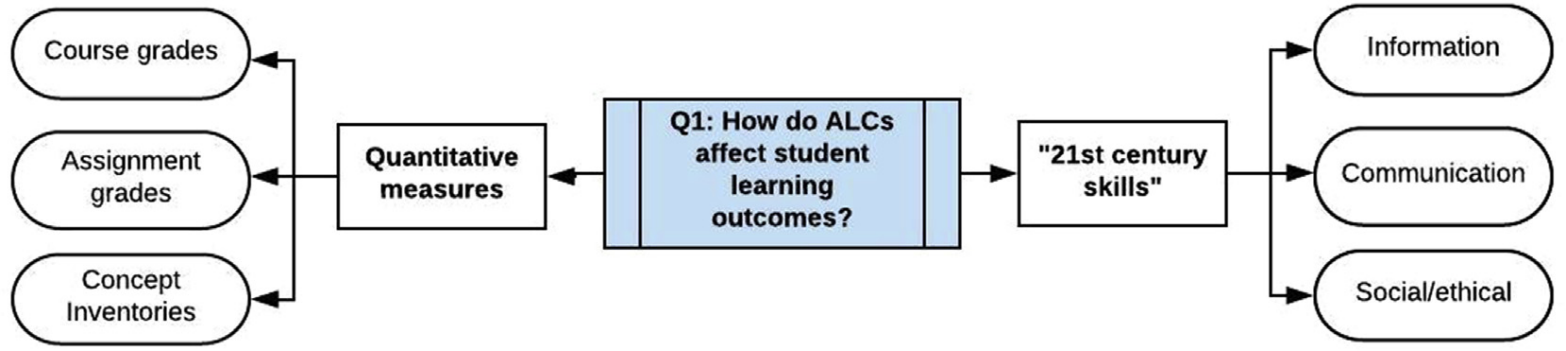
Research question 1.

**Figure 7:**
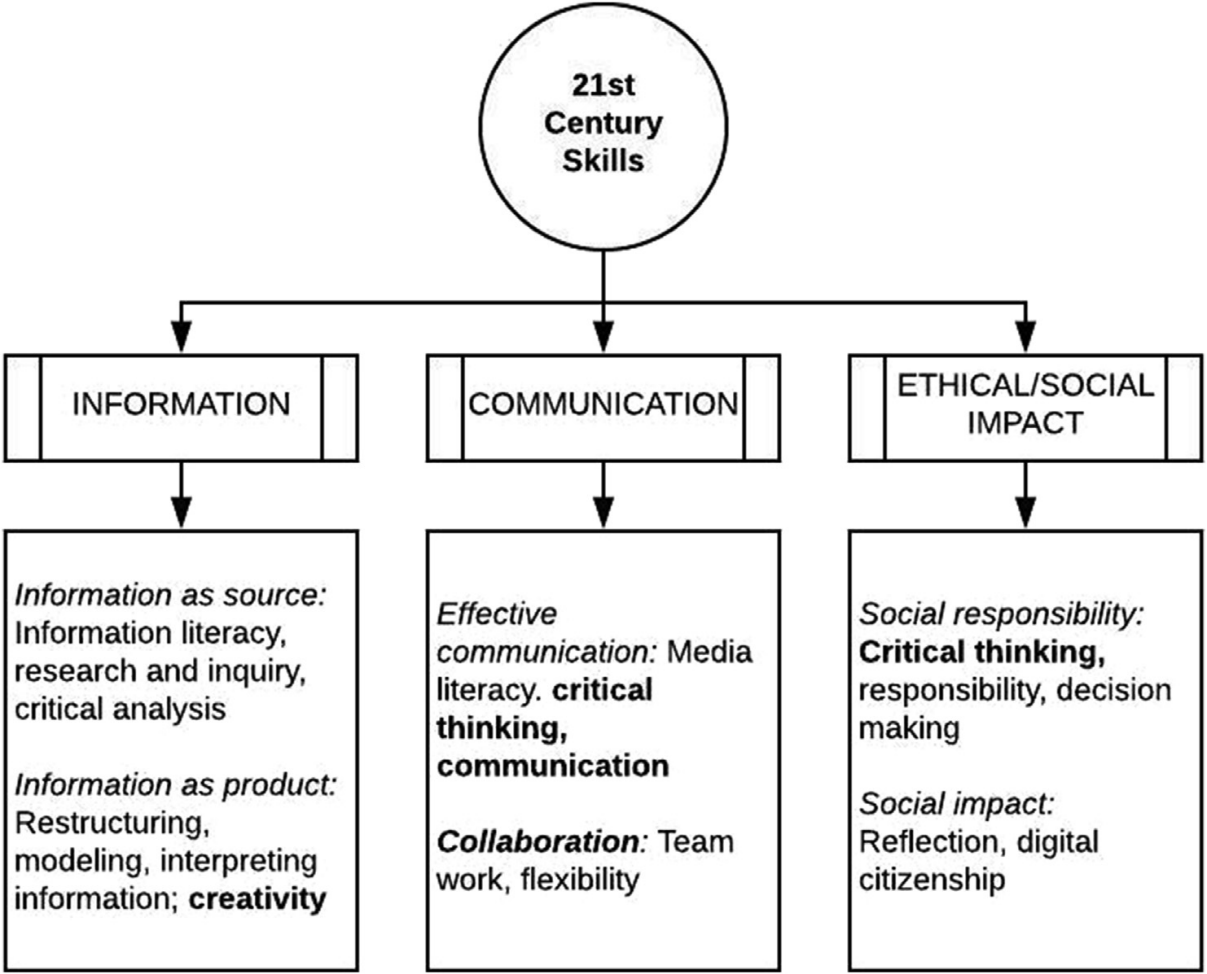
Framework for 21st century skills.

**Figure 8:**
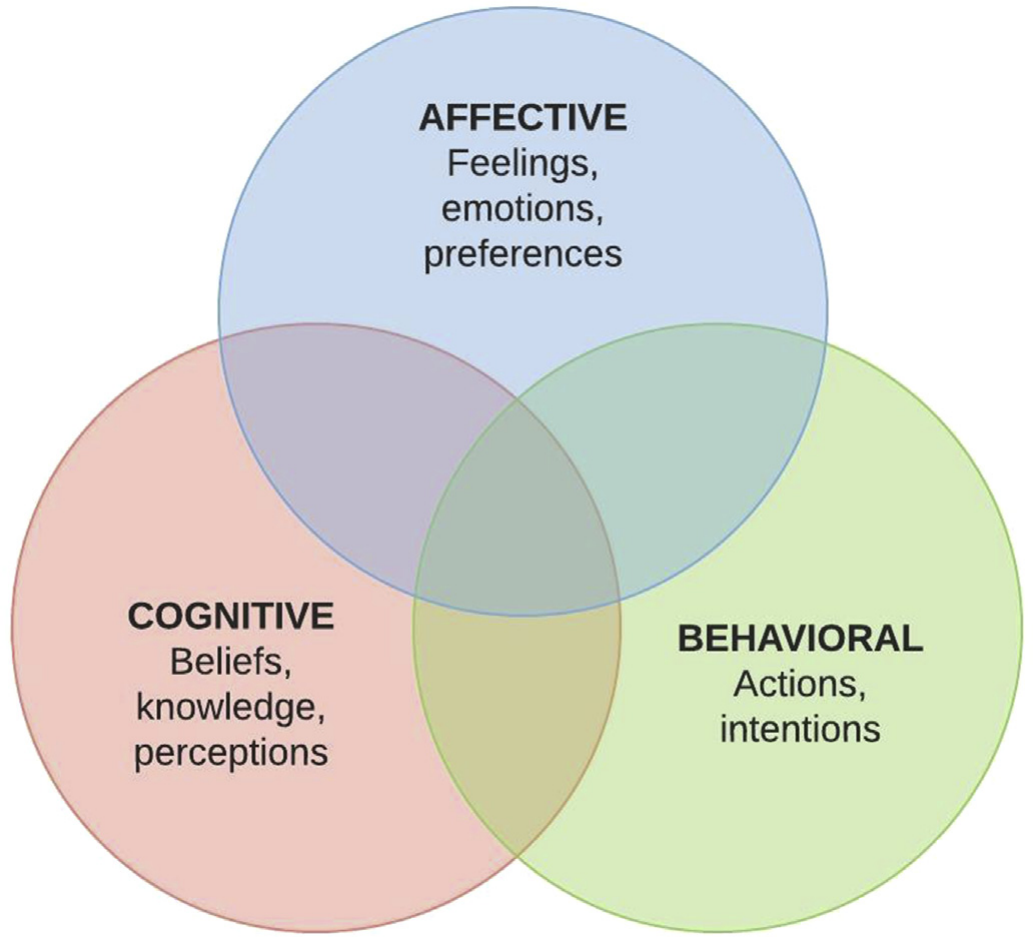
Framework for research question 2.

**Figure 9:**
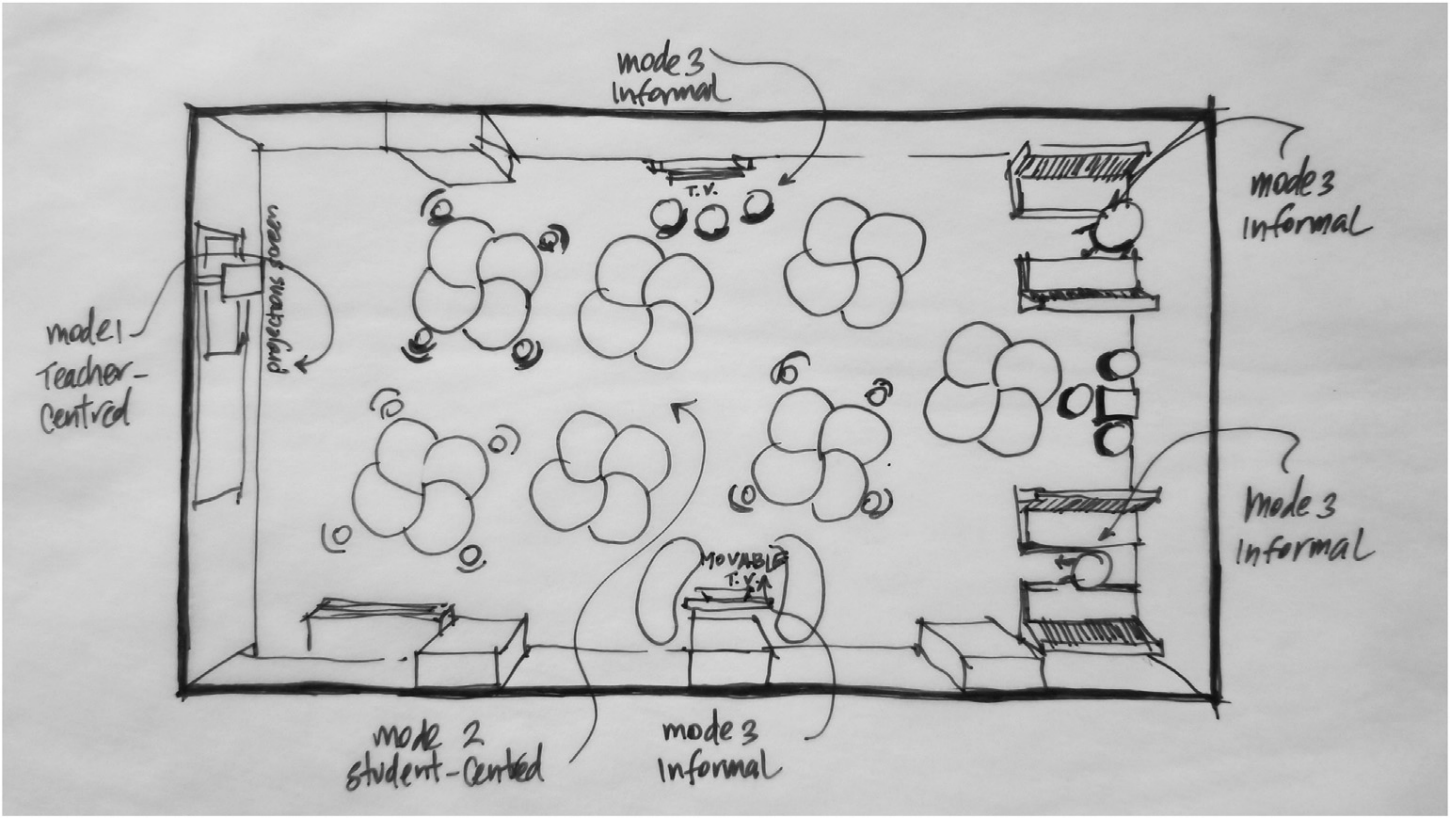
Next generation learning space, adapted from Byers and Imms (2014).

**Figure 10:**
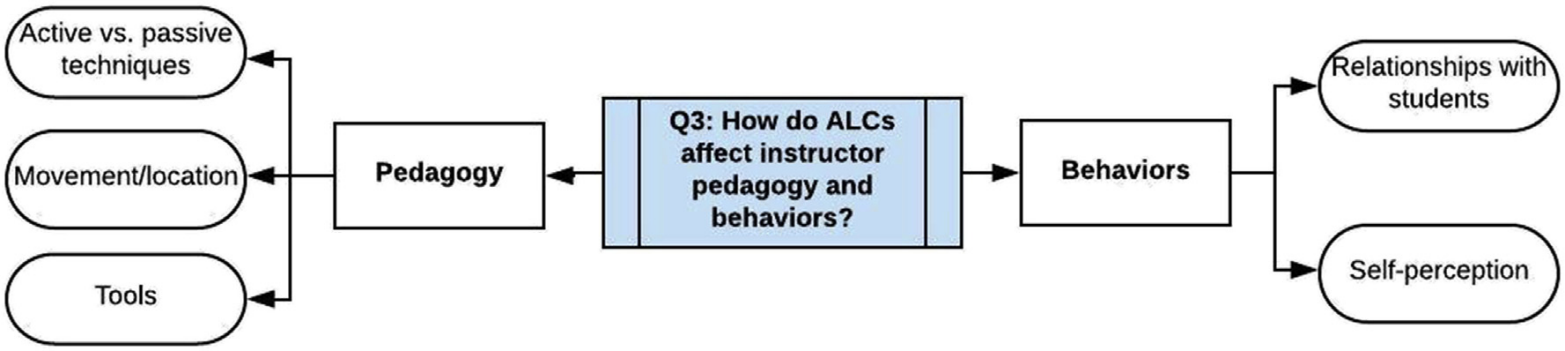
Research question 3.

**Figure 11:**
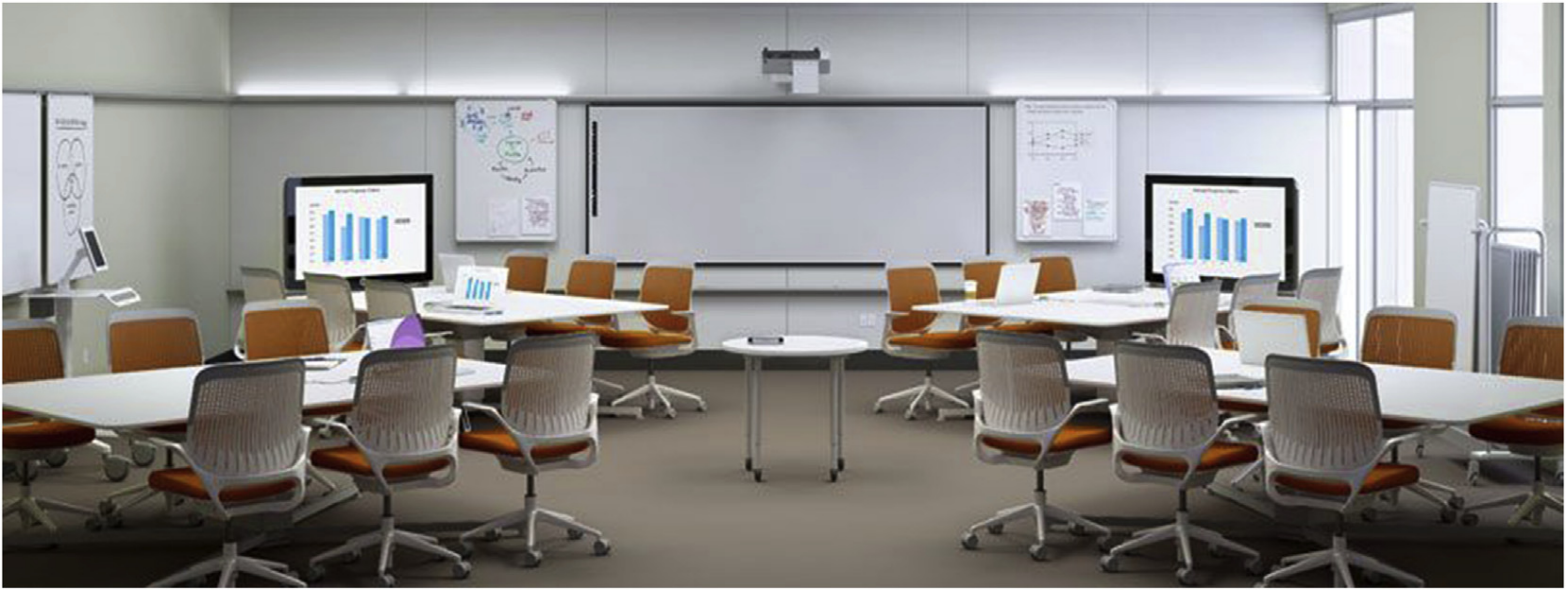
Steelcase LearnLab ALC.

**Figure 12:**
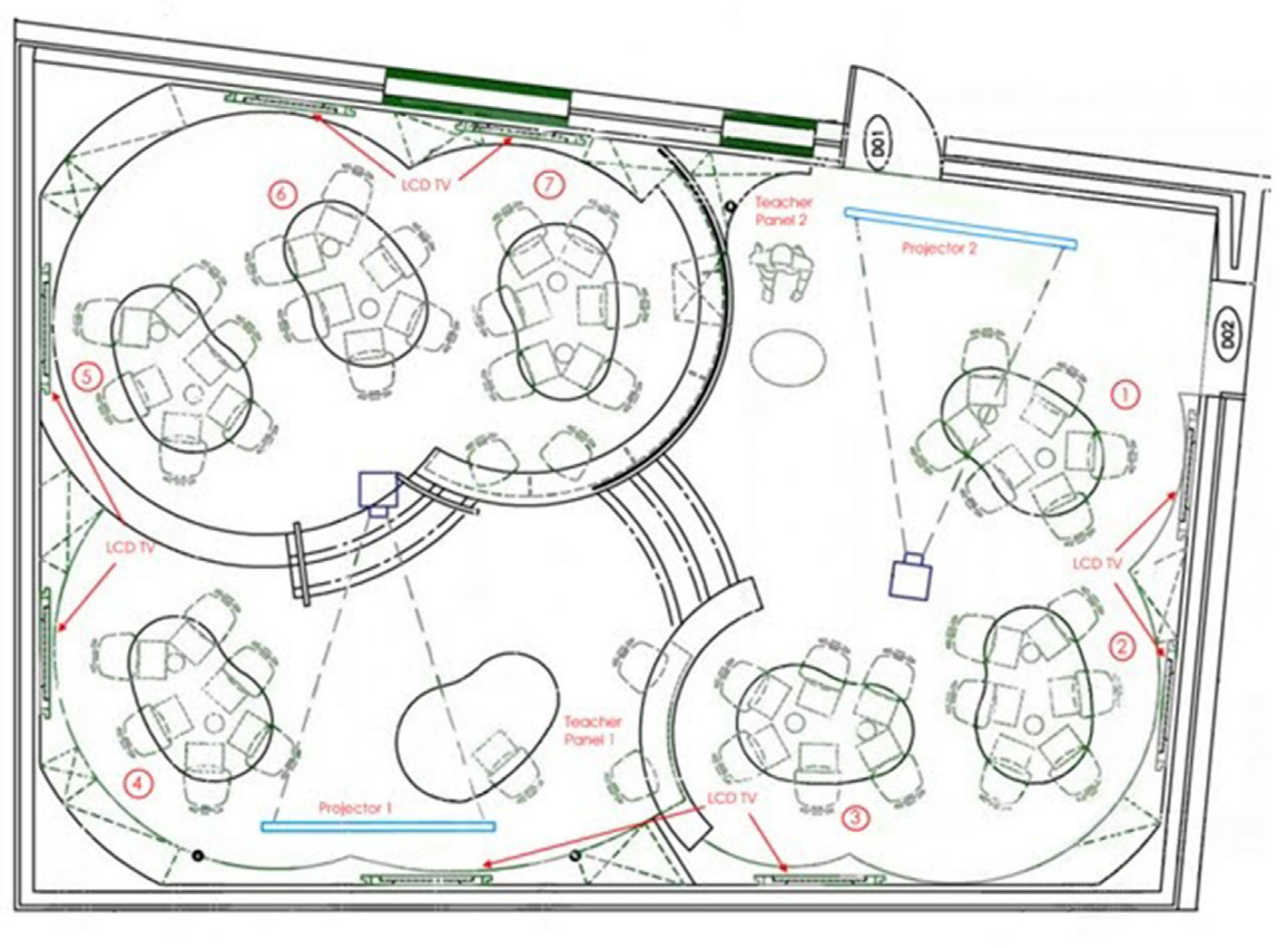
ALC with nonstandard layout.

**Figure 13:**
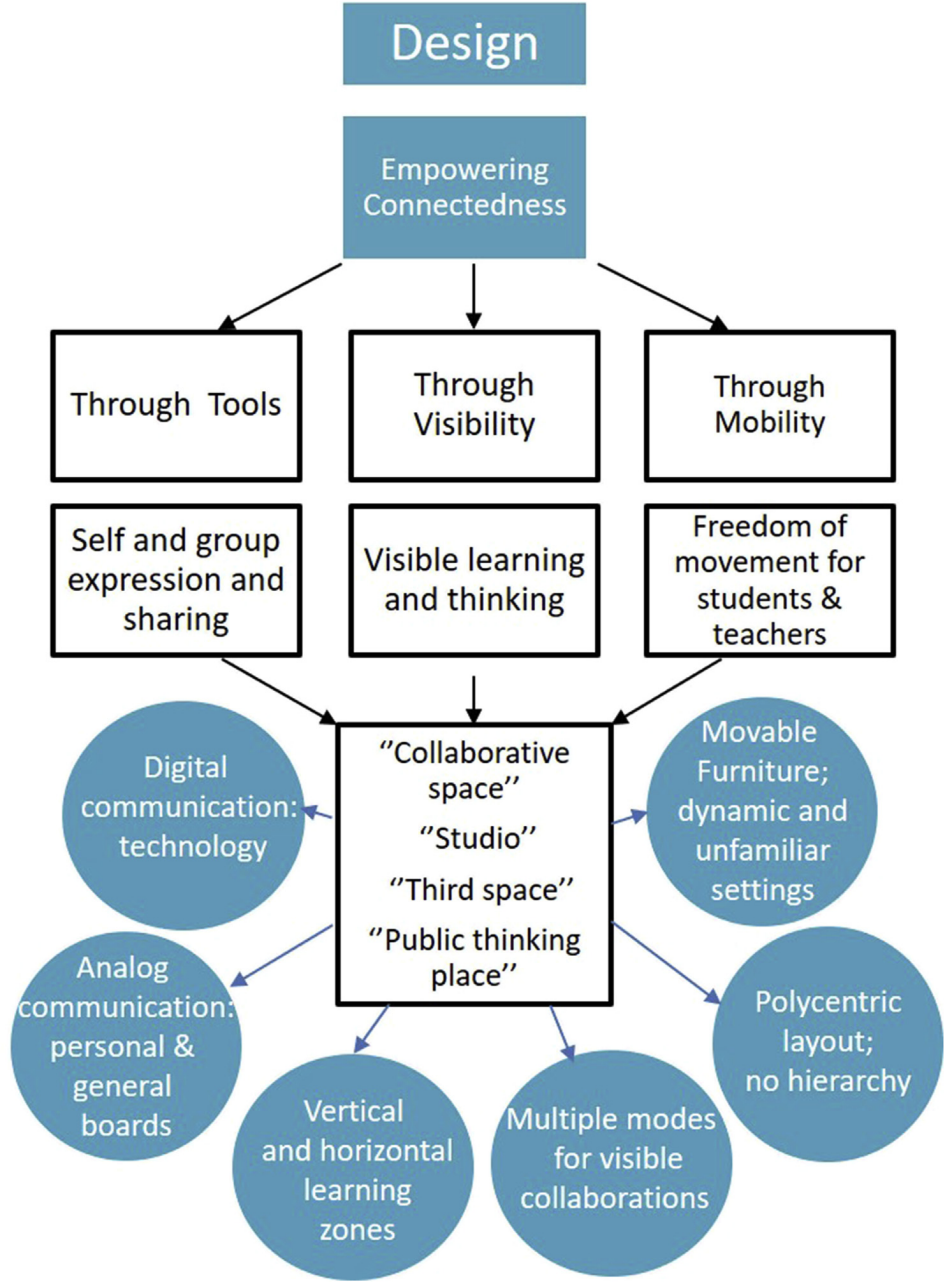
Design qualities and elements of ALCs.

**Figure 14:**
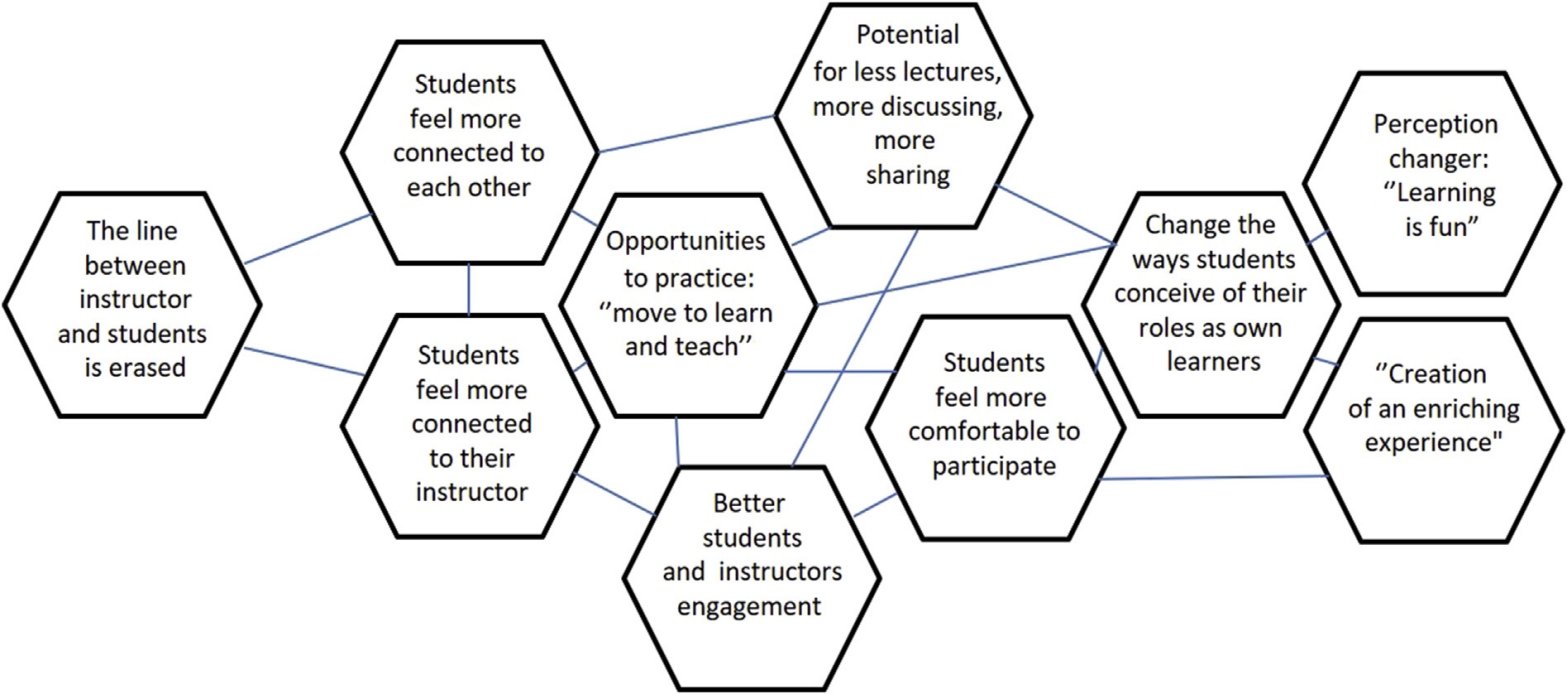
From the findings: the influence of ALCs on the new culture of learning.

*Active learning* is defined broadly to include any pedagogical method that involves students actively working on learning tasks and reflecting on their work, apart from watching, listening, and taking notes (Bonwell and Eison, 1991). Active learning has taken hold as a normative instructional practice in K12 and higher education institutions worldwide. Recent studies, such as the 2014 meta-analysis linking active learning pedagogies with dramatically reduced failure rates in university-level STEM courses (Freeman et al., 2014) have established that active learning drives increased student learning and engagement across disciplines, grade levels, and demographics (see Figures 1,2,3,4,5,6,7,8,9,10,11,12,13,14).

As schools, colleges, and universities increasingly seek to implement active learning, concerns about the learning spaces used for active learning have naturally arisen. Attempts to implement active learning pedagogies in spaces that are not attuned to the particular needs of active learning --- for example, large lecture halls with fixed seating --- have resulted in suboptimal results and often frustration among instructors and students alike. In an effort to link architectural design to best practices in active learning pedagogy, numerous instructors, school leaders, and architects have explored how learning spaces can be differently designed to support active learning and amplify its positive effects on student learning. The result is a category of learning spaces known as Active Learning Classrooms (ALCs)^1^.

While there is no universally accepted definition of an ALC, the spaces often described by this term have several common characteristics:

• ALCs are *classrooms*, that is, formal spaces in which learners convene for educational activities. We do not include less-formal learning spaces such as faculty offices, library study spaces, or “in-between” spaces located in hallways or foyers.
• ALCs include *deliberate architectural and design attributes that are specifically intended to promote active learning.* These typically include moveable furniture that can be reconfigured into a variety of different setups with ease, seating that places students in small groups, plentiful horizontal and/or vertical writing surfaces such as whiteboards, and easy access to learning technologies (including technological infrastructure such as power outlets).
• In particular, most ALCs have a *“polycentric” or “acentric” design* in which there is no clearly-defined front of the room by default. Rather, the instructor has a station which is either movable or located in an inconspicuous location so as not to attract attention; or perhaps there is no specific location for the instructor.
• Finally, ALCs typically provide *easy access to digital and analog tools for learning*, such as multiple digital projectors, tablet or laptop computers, wall-mounted and personal whiteboards, or classroom response systems.

### 1.2 A brief history of ALCs

Focused attempts to design learning spaces specifically attuned to active learning pedagogies date back to the 1990s. One of the earliest published efforts along these lines was the “studio physics” concept implemented at Rensselaer Polytechnic Institute (Wilson and Jennings, 2000; Wilson, 1994). A study of institutional costs of large lecture approaches to teaching introductory physics (which, as Wilson found, incurred large hidden expenses in the need for staffing labs and recitation sections) along with contemporary findings on the pedagogical shortcomings of lecture methods (Halloun and Hestenes, 1985) drove the design of classrooms seating 50 and 64 students each, with six-foot work tables holding two students each, arranged in concentric ovals around the center of the room.

The physics classes taught in these “studio” spaces were redesigned to focus on active lab-like activities done in groups of two students, seated so that they must turn away from the center of the room to work on their activities. Unlike traditional science courses that separate lecture and lab activities, studio physics courses conducted all learning activities in the same space.

Later, in an effort to adapt the studio physics concept to larger course sections, Robert Beichner and others at North Carolina State University redesigned their introductory physics courses using a combination of innovations in pedagogy, technology, and space in what they eventually deemed the Student Centered Active Learning Environment with Upside-down Pedagogy, or SCALE-UP (Beichner, 1999; Beichner et al., 2007). As with studio physics, SCALE-UP physics courses combined changes in pedagogy, technology, and space to refocus class activity on active learning in a combination of lecture, recitation, and lab tasks.

Students in SCALE-UP rooms are arranged at large circular tables seating nine students each (so they could work in three small groups of three students each, then combine into a larger group of nine) with affordances for technology such as multiple large projection screens for computer work and plentiful whiteboard space, and there was no clearly defined front of the room.

A similar initiative called Technology Enabled Active Learning (TEAL), also involving large polycentrically-designed rooms with round tables for active learning tasks, was undertaken at around the same time at MIT (Dori and Belcher, 2005) and a few years later at Iowa State University with the TILE (Transform, Interact, Learn, Engage) project (Van Horne, Murniati, Gaffney and Jesse, 2012).

While these ALC implementations were taking place in higher education, parallel investigations done by architects and designers were producing evidence against traditional lecture-focused learning spaces. One of the most prominent of these studies (Scott-Webber et al., 2000), simply titled “Higher Education Classrooms Fail to Meet Needs of Faculty and Students”, used a design based on the concept of proxemics (Hall, 1959) to conduct a qualitative study of faculty and students’ responses to traditional classrooms. The study found that key elements of the physical environment such as seating and noise control interfered with active learning and suppressed social activity and affective responses.

In addition to studies like that of Scott-Webber, Abraham, and Marini, research on ALCs began to accumulate shortly after their initial implementation, often conducted by the faculty members teaching in the space. Beichner's original report on the early implementation of SCALE-UP (Beichner, 1999) and the later, much-expanded follow-up study involving 16,000 students and multiple universities (Beichner et al., 2007) found remarkable improvements in student learning and engagement in SCALE-UP; these results were corroborated by research from the TEAL and TILE groups and later by others.

These promising results have helped fuel a surge in interest for ALCs among K12 and higher education institutions worldwide. EDUCAUSE named 2017 as “the year of the active learning classroom” (Brooks et al., 2017) and placed ALCs at the top of the list of higher education's top 10 strategic technologies for 2017 (Grajek, 2015). At Steelcase, Inc., a program to award 16 grants of up to $65,000 each to fund the construction of ALCs received over 1100 applications in 2018 from K12 and higher education institutions in North America. The interest in ALCs from many different educational sectors is strong and growing.

This interest has come with a common question: What evidence is there that active learning classrooms actually make a difference in the issues that matter to learners and educational institutions, such as student learning outcomes, student engagement and retention, and faculty adoption of research-based pedagogical practices? Put more simply, what assurances are there that institutions who expend large amounts of financial and other resources to install ALCs will reap positive benefits from doing so?

The purpose of this study is to examine the published literature up to the time of this writing to analyze and summarize the research on active learning classrooms, both in general and organized around several categories of importance to researchers and educational institutions alike. We seek to discern patterns of results among the studies we selected to discover the answers to the above questions about ALCs as well as best practices for using ALCs and opportunities for further research in this area.

## 2 Material and methods

### 2.1 Research questions

The main question that this study intends to investigate is: **What are the effects of the use of ALCs on student learning, faculty teaching, and institutional cultures?** Within this broad overall question, we will focus on four research questions:

*1. What effects do ALCs have on measurable metrics of student academic achievement?* Included in such metrics are measures such as exam scores, course grades, and learning gains on pre/post-test measures, along with data on the acquisition of “21st Century Skills”, which we will define using a framework (OCDE, 2009) which groups “21st Century Skills” into skills pertaining to information, communication, and ethical/social impact.

2. *What effects do ALCs have on student engagement?* Specifically, we examine results pertaining to affective, behavioral, and cognitive elements of the idea of “engagement” as well as results that cut across these categories.
3. *What effect do ALCs have on the pedagogical practices and behaviors of instructors?* In addition to their effects on students, we are also interested the effects of ALCs on the instructors who use them. Specifically, we are interested in how ALCs affect instructor attitudes toward and implementations of active learning, how ALCs influence faculty adoption of active learning pedagogies, and how the use of ALCs affects instructors’ general and environmental behavior.
4. *What specific design elements of ALCs contribute significantly to the above effects?* Finally, we seek to identify the critical elements of ALCs that contribute the most to their effects on student learning and instructor performance, including affordances and elements of design, architecture, and technology integration.

### 2.2 Identification of relevant studies

To find studies relevant to our research questions, we performed academic database queries using the databases ERIC (eric.ed.gov), ProQuest Education, LearnTechLib, Google Scholar, and Education Research Complete. We used the following terms for these queries:

• Active learning classroom
• Active learning space
• Active learning environment
• Active learning center

We discovered after our initial search that these terms are fraught with ambiguity. For example, many studies from searches on the term “active learning classroom” did not use the term “classroom” to refer to a physical space, but rather to a group of students (e.g. “My biology course is an active learning classroom” or “my classroom engages in active learning”). Similarly, the terms “space” and “environment” were sometimes used to refer to course design rather than physical space or environment (e.g., “I designed my course to be an active learning environment for students”). Therefore, the results of the database search were checked manually to ensure that the studies referred to physical spaces.

Three search queries were found to yield results that address our research questions: “active learning classroom”, “active learning space”, and “active learning center”. Those queries are hereafter referred to as Query 1, Query 2, and Query 3 respectively. Each query was performed on each of the databases listed above, with each search filtered to include only those studies that were peer-reviewed and published in scholarly journals. Ultimately, we used only the search results from the first three of these databases; Google Scholar lacked the tools to sufficiently narrow our search based on inclusion criteria (below), and Education Research Complete yielded only a few results, all of which were duplicated by the other databases.

We also noted that some studies on ALCs do not use the language in our queries. For example, the studies of Byers and Imms use the term “21st century classrooms” or “next generation learning spaces” which are not found elsewhere in the literature. Earlier studies do not use any special terminology referring to ALCs at all. Therefore, to capture these “false negatives” (studies known to pertain to ALCs but which did not appear in our searches), we used three known sources of information on ALCs: the literature review of Donna Gierdowski (2013), the studies referenced in Baepler et al. (2016), and the papers of Terry Byers and Wesley Imms along with studies found in the reference sections of the Byers and Imms papers.

### 2.3 Selection of studies

Performing Queries 1–3 on the ProQuest, LearnTechLib, and ERIC databases yielded 99, 35, and 117 results respectively, a large number of which were duplicates. Additionally, the reference section search using Brooks et al., Gierdowski, and the papers of Byers and Imms yielded 60 total studies with some duplicates. These results were filtered through the following inclusion criteria:

• We include only those studies that pertain to ALCs and not just learning spaces in general.
• We include only those studies that examine an intervention involving the introduction of an ALC to an actual student learning situation such as a class or multiple sections of a class.
• We include only those studies that collect empirical qualitative or quantitative data on the effects of the intervention towards one or more of our five research questions, with sufficiently scientific methodology and in sufficient quantities to merit rigorous analysis.

For example, we excluded studies that examined the effects of space on student learning but which did not specifically involve ALCs (for example, Meyers-Levy and Zhu, 2007). We also excluded studies that examined the nature, architecture, or properties of ALCs but did not perform an intervention on students or which did not collect qualitative or quantitative data. We also excluded studies that focused on ALCs and did collect some data, but only collected anecdotal evidence or evidence from only a very small number of respondents.

After removing duplicate search results and filtering out studies that did not meet the inclusion criteria, the total number of distinct studies from the database queries was 21. We also found 60 studies in the references in Brooks et al., Gierdowski, and Byers and Imms; after removing duplicate entries and applying the inclusion criteria, the results from the non-database references was 55. The database and reference section results were then combined and duplicates removed, and a final round of the inclusion criteria applied, to arrive at a final count of 37 studies.

### 2.4 Data charting and collation

Using the studies identified through our searches and inclusion criteria, we read each article carefully and compiled notes on the setup, methods, and results from each. We summarized each of these studies in a table (below) with entries for the authors and year of the study; the context (primary education, higher education, etc.) and location of the study; the type of intervention done; the design of the study; and a summary of the results. A summary of the results can be found in the table located in Appendix A. An electronic version of this table, suitable for downloading and data analysis, can be found at the following link: http://bit.ly/ALC-litreview-table.

### 2.5 Overall characteristics of the results

All of the studies identified through our search took place with actual students in educational settings (as opposed to experimental studies done in controlled laboratory-like settings). The majority of these (32 out of 37) were conducted in higher education, with university courses and university students. Of the remaining studies, two were conducted in secondary education settings; two more were conducted using grade and age levels in late primary/early secondary education settings (specifically, students in years 7 and 8 of formal schooling); and one study conducted in a primary education setting.

In addition, 27 of the 37 studies were conducted in the United States of America (and all of these were done in higher education settings). Roughly half (5 out of 11) of the remaining studies were conducted in Australia in research connected with Terry Byers and Wesley Imms; and the rest took place in various other geographic and cultural contexts.

Finally, we note that most of the research identified in this study took place between 2012 and 2016:

### 2.6 Methodological characteristics of the studies

The methodologies employed by the 37 studies that met our inclusion criteria varied widely. We have summarized the design and results of each of the studies in Appendix A: Summary Table of Results where the reader can find abbreviated information on the educational context, type of intervention, study design (including sample sizes and type of design), which of our research questions were addressed by each study, and results of each of the studies in this review. Because of the wide variation in the studies we considered, a few notes are in order:

• The sample sizes used in the studies were sometimes not specified in the studies themselves, or different populations and samples were used within single studies. For example, some studies did not list their population sizes at all (Connolly and Lampe, 2016), while others discussed the number of *classes* that were studied but not the number of students (Hyun et al., 2017), while still others studied both students and faculty and give sample sizes for both.
• We have attempted to note which of our four research questions were addressed by each study in Appendix A, and sum up how many studies address each research question in Section 3 (Results). However, it was not clear in some cases whether a particular research question was being addressed by a particular study. In particular, all of the studies in this review in some sense address research question 4 (Which elements of design most affect student and instructor outcomes?) but only a few of those studies address this question directly as an intentional element of the research design. In other cases, the line between student learning outcomes and student engagement in the design and results of a study was blurry. Therefore our estimation of which questions were addressed by each study is a judgment call.

More specific comments on the methodologies of the studies involved will be given in Section 4 (Discussion).

## 3 Results

### 3.1 What effects do ALCs have on measurable metrics of student academic achievement?

In this section we address the research question, *What effects do ALCs have on measurable metrics of student academic achievement?* We separate these metrics of academic achievement into two categories: grades and other quantitative outcomes of learning that are focused on content mastery, and so-called “21st century skills” focused on non-content academic skills such as the management of information. Of the 37 studies in this review, we found 10 that address this particular question.

#### 3.1.1 Quantitative measures of student learning

Many of the studies in this review focused on quantitative measures of student learning, generally grouped into three categories: grades on individual assignments (e.g. final exams) or groups of assignments, course grades, and learning gains on pre-versus post-test measures of concept inventories. We now summarize those results using each of those categories.

The earliest study in this review is by Oliver-Hoyo et al. (2004) and focuses on assignment grades. The researchers used an implementation of the SCALE-UP model in an introductory university chemistry class that employed the “concept Advancement through chemistry Lab-Lecture" (cAcL_2_) pedagogical model in which lecture and laboratory components are done together rather than separately. The study compared the grade outcomes on various assignments for students in the ALC section of the course with those taken by similar students in a traditional setting in which lecture and labs were conducted separately. They found no statistically significant differences between the sections when taking averages across exams for each section. However, the exam × class interaction was significant; students in the ALC section scored significantly higher on the second and fourth exams than the students in the traditional section. There were no statistically significant differences on the remaining exams, although the bottom 25% of students in the ALC section scored significantly higher than the bottom 25% of students in the traditional section on the last three exams. This study illustrates two results that appear elsewhere in this review: (1) ALC sections of courses often require time for students to acclimate to the space before positive results are seen, and (2) ALC sections tend to show the strongest learning gains among the lowest-performing students.

In a study with a different focus, Muthyala and Wei (2013) compared the exam scores of students in two different layouts of ALCs, rather than ALC vs. traditional space. The two layouts, “Spoke” (students seated at tables radiating outward from the center of the room) and “Node” (students seated in moveable chairs in clusters of four; not to be confused with the Steelcase seating product of the same name), were used in two sections of an introductory organic chemistry class and two sections of a more advanced organic chemistry class, and student performance on exams between sections of the same course were compared. The study found no statistically significant differences on a composite “summative evaluation” score composed of exam scores, the final exam, and the course grade between the ALC and traditional sections. However, there was a non-significant but “noticeable” difference in the first exam, with the “Node” layout performing better than the “Spoke” layout, but on subsequent exams this was reversed, with “Spoke” having higher exam grades. The authors posit that the differences in the first exam score are due to the similarity of “Node” to more traditional layouts, but then as students become more comfortable with collaboration, they become more attuned to the affordances of the “Spoke” layout.

A study by Brooks and Solheim (2014) examined both the course grade and individual assignment grades in a SCALE-UP environment compared to a traditional space, using two sections of a university-level personal finance course. They found statistically significant differences in the course grades, with the ALC students scoring higher than the traditional section (85.5% versus 81.8%). In examining whether the differences in course grades might be an artifact of a single assignment (e.g. higher participation rates in the ALC section) rather than systematic improvement, average scores in each major group of assignments (attendance, a financial planner project, case studies, the final exam, and quizzes) were compared. The students in the ALC section scored significantly higher in each group than did students in the traditional section, at either *p* < 0.001 or *p* < 0.0001 levels.

Shifting focus to course grades alone, Brooks and Solheim (2014) also examined the differences in course grades between students in the quartiles of each section and found statistically significant difference in each quartile, with ALC students having higher course grades than their traditional section counterparts in every quartile. The grade differences in the first, second, and third quartiles were significant at the *p* < 0.0001 level, while the grade differences in the fourth quartile were significant but only at the *p* < 0.05 level.

Several other studies give results on the course grades of students in ALCs, either by comparing course grades between ALC and traditional sections of courses or by comparing proportions of students who fail courses in an ALC versus a traditional section. Beichner et al. (2007) report that the pilot implementation of SCALE-UP across several institutions resulted in a 40–60% reduction in the failure rates of students in introductory university physics courses compared to students in the same course in a traditional space. The same study examined “failure ratios” in introductory physics courses taught in SCALE-UP versus traditional environments; this term is defined to be the percentage of students who failed the course taught in a traditional space divided by the percentage of students who failed taught in the SCALE-UP space. Failure ratios were computed for students in various ethnic and gender groups, and the results ranged between 2.1 for Asian-American students to 4.7 for female students. The failure ratio for Hispanic students could not be computed because none of the Hispanic students failed the SCALE-UP courses.

Three related studies (Brooks, 2011; Cotner et al., 2013; Walker et al., 2011) again studied the course grades of students in ALC sections of courses versus those of students in different sections of the same course taught under similar conditions but in a traditional space. In each of these studies, students in the ALC section were found to have significantly lower scores on their American College Test (ACT) scores than students in the corresponding traditional section. In each study, a regression model showed a statistically significant correlation between ACT score and final course grade based on past data. All three studies found no statistically significant difference between the final course grades of students in an ALC section versus those in the traditional section --- even though the regression model predicted the ALC students would have significantly lower grades. By contrast, students in the traditional sections obtained course grades in line with what the regression model predicted. The authors conclude that the ALC environment served as a catalyst for students to perform better than reliable statistical models would predict.

One study (Baepler et al., 2014) examined the use of an ALC in a large (350-student) undergraduate general chemistry class by splitting the class into three cohorts of roughly 100–115 students each, and having each cohort meet in a 117-student SCALE-UP room once per week, instead of the entire class meeting in a traditional space three times per week. The remaining two days per week of activities for each cohort were spent working through online activities. Two sections of the course were blended in this way, and a control section was kept intact in a traditional space. Grade and demographic data were collected as well as two examinations developed by the American Chemical Society and a single exam generated by the instructors of the class. The researchers found moderate performance differences between one of the experimental blended groups and the control group, and no statistically significant difference between the other blended group and the control. After controlling for demographic and aptitude variables, however, the blended approach yielded results that were the same or better than the traditional approach.

Finally, we look at studies that examine normalized gains on concept inventories. The normalized gain (Coletta and Phillips, 2005) is a numerical measure computed using the outcomes of a pre-test and a post-test administration of an assessment (in this case, a concept inventory). The normalized gain is defined as the ratio of actual student gains between the pre-test and post-test, to the theoretical gain:

$$\langle g\rangle =\frac{\left\langle post\rangle -\langle pre\right\rangle }{100-\langle pre\rangle }$$

The normalized gain is therefore a number between 0 and 1 inclusively, with a higher score indicating greater improvement. A score of 0 means the pre- and post-test scores were the same, indicating no improvement; a score of 1 indicates a perfect score on the post-test, i.e. the maximum amount of improvement has occurred. Typically, normalized gain is computed using for entire classes using class averages, rather than one student at a time.

Beichner et al. (2007) examined normalized gains across students in university physics courses at several different sites using different concept inventory instruments. In one setting, students in a SCALE-UP section of an electricity and magnetism course showed significantly higher normalized gains on the Conceptual Survey of Electricity and Magnetism, the Electric Circuit Conceptual Evaluation, and the MIT Electricity and Magnetism Test, than students in a traditional setting, with the largest differences in normalized gains found among students in the top one-third of the courses. Students in SCALE-UP sections of the mechanics portion of an introductory physics course showed significantly higher normalized gains on the Force Concept Inventory compared to students in a traditional section, with the traditional section obtaining normalized gains of 0.204 and 0.176 for regular and honors sections of the courses (respectively) compared with normalized gains of 0.483 and 0.477 for regular and honors sections of the SCALE-UP course.

#### 3.1.2 “21st century skills”

ALCs are generally considered to have an effect not only on content-oriented measures of student learning but also on proficiencies often called “21st century skills”. We examined the studies in this review for evidence of the impact of ALCs on “21st century skills” as a form of measurable student learning outcome, separate from and orthogonal to content-based measures of achievement.

Despite their frequent use in current discourse about education, there is considerable disagreement on what constitutes “21st century skills”. A common rubric for these skills is “The Four C's” --- communication, creativity, collaboration, and critical thinking (Framework for 21st Century Learning - P21, 2019). However, there is in fact no standard operationalization of this term. In many contexts, “21st century skills” refers to a set of competencies associated with processing and communicating information. In others, it refers to items more properly characterized as behaviors, such as motivation, perseverance or “grit”, or flexibility. Ananiadou and Claro (2009) point out that lists of “21st century skills” is dependent on culture, with different world cultures placing different values on individual skills.

To provide some standardization for interpreting the results of studies that purport to examine “21st century skills”, we will adopt the framework presented by the Organization for Economic Cooperation and Development (OCDE, 2009), which categorizes “21st century skills” into three dimensions:

• **Information**: This dimension is subdivided into *information as source*, which includes skills pertaining to finding and organizing information such as information literacy, research and inquiry, and critical analysis; and *information as product*, which includes skills at restructuring, modeling, and developing one's own ideas about the meaning of information. The OECD framework includes “creativity” and “innovation” in this sub-dimension. Although not explicitly included in the OECD framework, we include general “problem solving skills” in this dimension as well, since this concept occurs frequently in other discussions of “21st century skills” and is a combination of working with information as a source and as a product.
• **Communication**: This dimension is also subdivided, into *effective communication,* which includes skills in critical thinking and interpersonal communication; and *collaboration and virtual interaction*, including skills such as teamwork and flexibility.
• **Ethical and social impact**: As with the other two, this dimension is subdivided into *social responsibility*, including skills in critical thinking (seen as distinct from critical thinking in the communication dimension, although with overlap between the two), personal responsibility and decision making, and *social impact* which includes such skills as reflection and “digital citizenship”.

Note that this framework includes all of the “Four C's”, although “critical thinking” is mapped to two different dimensions. Critical thinking in this framework can refer to two skills: The mindful use of information when engaging in discourse with another (Communication), and the analysis of information to make decisions (Ethical/Social Impact).

We first highlight a study by Nissim et al. (2016) because it addresses a wide range of 21st century skills in an ALC used by pre-service teachers in Israel. Students were given the Active Learning Post Occupancy Evaluation (AL-POE) (Scott-Webber et al., 2014), which contains items in several areas related to what the authors describe as “21st century skills”. For the purposes of this review, we will only focus on those skills that fit the OECD framework given above. The study found that:

• 100% of students and approximately 95% of instructors reported moderate, high, or exceptional increases in the ability to be creative in the ALC.
• When asked to rate both their old/traditional classrooms and their ALCs as “adequate” in several different factors, student ratings were significantly greater for ALC's (at p < 0.002 significance) for the following areas: collaboration, active involvement, and in-class feedback, for both pedagogical practices and pedagogical solutions. (Several other factors that do not fit into the OECD 21st Century Skills framework were similarly highly rated.)

Other studies in the review have more targeted results in each of the three dimensions of the OECD framework:

• *Information dimension*: The SCALE-UP report from Beichner et al. (2007) reports not only significant improvements on student learning outcomes as reported in the previous section, but also reports that students’ “[a]bility to solve problems is as good or better” in the SCALE-UP environment as in a traditional college physics setting. The authors appear to extrapolate this observation from the data on physics content outcomes, rather than collecting additional data on general problem-solving skills. A study by Rands and Gansemer-Topf (2017) reports students using tools afforded by ALCs to engage spontaneously in visualizing information, even when not explicitly instructed to do so. Finally, in a very large scale study (Chiu and Cheng, 2016) involving n = 35953 students in general education courses taught in ALCs in a Hong Kong university, students reported significantly better learning experiences overall and a significantly higher rating for “encouragement to be creative and innovative” when compared to the same course taught in a traditional space.
• *Communication dimension*: Some studies of ALCs report students, instructors, or both stating that the ALC “affords” improvements in communication and collaboration (Connolly and Lampe, 2016; Rands and Gansemer-Topf, 2017) --- that is, the respondents in those studies imply that communication and collaboration take place in an ALC at a level that would not be present in a traditional classroom space. (We note that data reported in Connolly and Lampe (2016) are quite limited; survey results were not reported, and only three individual responses from student focus groups are given.). Additionally, Cotner et al. (2013), in an observational study, report that students in an ALC objectively engage in more group activity and collaboration than in a traditional classroom space. Finally, students in the study by Byers and Imms (Byers and Imms, 2016) report significantly more interactivity and collaboration take place in an ALC than in a traditional space.
• *Ethical/social impact dimension*: The only study in the review that reports data on this dimension of 21st Century Skills is (Chen, 2014), in which 28 fourth-year students in psychology were instructed in an ALC and given the Active Open-Minded Thinking (AOT) questionnaire (Stanovich and West, 1997) as a pre- and post-test measure. Chen (2014) explains that AOT is “a person's ability to actively reflect on his/her thinking, actively seek and process information that contradicts his/her beliefs, and be willing to alter his/her mindset after carefully considering opposing beliefs” (pp. 173–174). Chen found that students AOT generally increased over the term, although increases among students with high initial AOT were negligible.

### 3.2 What effects do ALCs have on student engagement?

The second research question we investigated is, *What effects do ALCs have on student engagement?* This dimension of the student learning experience is connected to, but quite distinct from the effects noted in the previous section on student learning outcomes. A large number of the studies in this review (31 out of 37 in our estimation) targeted student engagement, and there has been a strong and sustained interest in “engaged learning” at all levels of discussion on teaching and learning. The results of the studies in this review that specifically target “engagement” are therefore numerous, widely varied, and of potential use to many school leaders and educators.

However, we must first come to terms with the meaning of the word “engagement” as it relates to learning. Unfortunately, despite the wide usage of “engagement” in academic and popular discourse, the term remains poorly operationalized. At times, it is defined in terms that describe certain student activities and behaviors, while at others it describes what parents, teachers, and school leaders do to elicit those activities and behaviors. Even when confined just to students, “engagement” can sometimes refer to feelings and emotions; in other cases, to the things students do, say, or intend; in still other situations, to beliefs or perceptions that students hold; or a combination of these, often undifferentiated between the aforementioned aspects of “engagement”. There is no consensus on a definition of “engagement” from which to conduct a rigorous analysis of research on the subject; and given the wide array of meanings of this term in actual use, such a definition is unlikely to come to light in the near future.

Therefore, in this literature review, we will not attempt to rectify the various concepts of “engagement” that permeate discussions of teaching and learning. We will only give some background on the origins of the term, and then state a model for “engagement” that we will use to parse and frame the various results in this review's studies that pertain to engagement.

As Axelson and Flick (2010) point out, the term “engage” originally derives from a Norman word meaning “pledge”, so that to “engage” originally meant to enter into a binding obligation to another through oaths or laws (The term “mortgage” derives from the same root and has a similar connotation.) The modern usage of the word is similar: To be “engaged” means to pledge oneself to a binding involvement with something or someone, much like the usage of this term in the context of a pledge to be married. In an academic setting, “engagement” can therefore be understood as a state of committed involvement, in an activity into which we have entered willingly and with the intention to complete, and in which “we are entirely *present* and not somewhere else” (Axelson & Flick, p. 40, emphasis in original).

The concept of *involvement* speaks to a more modern origin of the concept of academic engagement. Alexander Astin's research on student involvement in the 1980's (Astin, 1999) is considered by many scholars to be the forerunner of the modern notion of “engagement”. Astin's theory of involvement “refers to the investment of physical and psychological energy in various objects” (Astin (1999), p. 519); occurs along a continuum with different students contributing different levels of investment in the same objects; and has both quantitative and qualitative aspects. Astin further ties the quantity of student learning and development as well as the effectiveness of educational policy to the quality and quantity of student involvement.

In later explorations of “engagement”, the qualitative and quantitative features of “involvement” tend to take on three distinct, yet strongly overlapping categories: *affective* forms of engagement, which pertain to feelings and emotions attached to student involvement (or the lack thereof); *behavioral* evidence of engagement, describing student actions that indicate involvement; and *cognitive* engagement, referring to beliefs, knowledge, and perceptions connected to involvement.

We note that this tripartite framework aligns well with the “ABC” (Affective/Behavioral/Cognitive) model of attitude promulgated by Eagly and Chaiken (for example (Eagly and Chaiken, 1998),) and others. Indeed, the modern concept of student engagement is closely tied to student attitudes about learning, and thus this framework for engagement is appropriate for the results found in this review.

As we begin to examine the studies on ALCs and engagement, we will use the above framework for engagement and an understanding of the etymology of the word to guide our understanding of the results. In those results, we identified two major groups of studies about engagement: Those that focused on only one of the three particular facets of engagement in our model, and those that addressed more than one form of engagement.

#### 3.2.1 General or undifferentiated results about engagement

We begin by examining studies whose focus on engagement either did not differentiate between the three areas of engagement in our model, or did recognize the different areas but examined more than one of them.

We first highlight two results from our review that explicitly connect the elements of ALCs to various facets of student engagement. These center on the use of the Active Learning Post-Occupancy Evaluation (AL-POE) instrument developed by Scott-Webber et al. (2014) to gather quantitative data about the impact of ALC's on engagement.

Scott-Webber et al. (2014) developed the AL-POE to measure student engagement, as a behavioral indicator using twelve parameters:

1) “…collaboration
2) focus
3) active involvement
4) opportunity to engage
5) repeated exposure to the material through multiple means
6) in-class feedback
7) real-life scenarios
8) ability to engage
9) physical movement
10) stimulation
11) feeling comfortable to participate
12) creation of enriching experience” (p. 4).

The result was an instrument that sought to measure the effect of evidence-based, intentionally designed solution interventions on student engagement in the formal learning place. The AL-POE aimed to explore the comparison between old/pre (row-by-column seating) environment and new/post intentionally designed environment by identified student engagement factors. Respondents to the AL-POE are asked to rate the adequacy of the old learning space and the new learning space on factors connected to the twelve parameters above.

In Scott-Webber et al. (2014), three different universities participated in the study that included 124 students and educators in three types of new environments where a combination of intentionally designed active learning furniture designed by Steelcase, Inc. was included. The design of the study was a comparison of the experience between a traditional classroom and the new experience in the active learning classrooms. The results showed statistically significant differences (p < 0.001) on all twelve factors between the behavior in a traditional classroom and the behavior in the ALC. A later study by Scott-Webber and others (Scott-Webber et al., 2017), while not included in our main results because it does not involve ALCs and therefore does not meet our inclusion criteria, verifies that active learning techniques and physical movement are significant indicators of student engagement and that the design of the built environment can have an effect on the connection between pedagogy and engagement.

A later study that also used the AL-POE (Nissim et al., 2016) administered the instrument to 87 students and 15 instructors in an Israeli teacher training college and used the results to examine self-ratings of whether ALCs enhanced their creativity, motivation, engagement in general, and perceived ability to achieve a higher grade. Over 90% of instructors and 80% of students reported moderate, high, or exceptional increases in these factors in moving from a traditional space to an ALC. Further, an “overall engagement score” was calculated from the AL-POE results. The overall engagement scores for both practices and solutions in the ALC space were statistically significantly higher (at the p < 0.0001 level) than those scores in the traditional space.

A similar line of inquiry about engagement, in which students are exposed to both traditional and ALC spaces and then asked to rate each space numerically using various parameters, can be found in the papers of Australian researchers Terry Byers and Wesley Imms and their co-investigators. The studies of Byers and Imms share a common methodology, namely a single-subject research design (SSRD). Under this design, a single cohort of students (and sometimes instructors) experiences both a “control” condition (learning in a traditional space) and then an “experimental” condition (learning in an ALC), with all other variables such as pedagogy and technology held relatively constant. In most of Byers and Imms’ work, the students return to the traditional space for a third phase, and in some studies the students return again to the ALC for a fourth phase. At the beginning and end of each phase, students are given a survey that measures self-reported data on various aspects of the learning process, resulting in between four and eight sets of comparative data. A feature of Byers and Imms’ design is that major confounding variables such as pedagogical techniques and the use of technology are controlled for, allowing space to be the only true variable in the study and hence create a much stronger connection between the variance of space and the results of the survey. Byers and Imms, in fact, claim a truly causal link between space and their results.

In 2014, Byers and Imms conducted a study that produced two separate papers (Byers and Imms, 2014; Byers et al., 2014) connecting ALCs to improvements in engagement. The study involved 164 students, grouped into six classes comprised of both year 7 and year 8 students, in three buildings in a primary/secondary religious school in buildings constructed between 1940 and 1960. Two groups of classroom spaces were involved in the study: a traditional space, and a “next generation learning space” with a polycentric layout allowing for three different teaching and learning modalities to exist in the same space. Students learned first in the traditional space and then in the ALC, taking pre- and post-measure surveys in each phase.

The first paper (Byers and Imms, 2014) focused on survey results pertaining to students’ perceptions of the effectiveness of using one-to-one technology in their learning. The researchers found statistically significant higher ratings of ALCs versus traditional spaces in the areas of "positive influence” (i.e. whether students perceived the use of technology as having a positive influence on their learning), "effectiveness", and "flexibility", in 11 out of the 18 possible combinations of student class and question type. Furthermore, a teacher focus group was held to follow up the surveys; in the focus group, teachers reported the ALCs afforded higher levels of student collaboration.

Whereas Byers and Imms (2014) reports on somewhat narrow facets of student engagement, the second set of results from this study (Byers et al., 2014) takes a broader view, reporting back on survey results that target engagement generally speaking. The researchers found that the ALC phase of the study generated significantly higher ratings on "student learning experience” and "student engagement” in all but one class’ response to one group of questions, and the effect sizes suggest "an improvement of 1–2 standard deviations from the traditional classroom mean” (p. 8).

Another study from Byers and Imms (Byers and Imms, 2016) involved 94 year 4 students in an Australian primary school (We note that this is the only study identified in the review focusing specifically on primary schools.) The cohort attended class sessions in two spaces: a traditional space featuring rows of desks facing a well-defined front of the room, and an ALC with a polycentric layout and adjustable-height tables. Both spaces were outfitted with technology such as interactive whiteboards, wireless internet connectivity, and tablet computers. Following the pattern of single-subject research design, students spent a period of time in the traditional space, then a similar period in the ALC, and then moved back to the traditional space. At the beginning and end of each phase, students were given the Linking Technology Pedagogy and Space (LTPS) survey instrument which poses 10 items in three domains regarding students’ perceptions of technology, learning experiences, and engagement. (According to our model, all three of these domains can be considered “engagement”.) Students were grouped into four classes, giving 40 possible combinations of class and survey item. The researchers found statistically significantly higher ratings for the ALC in 22 out of 40 of those combinations spanning all three domains, with significant results in all four classes on items pertaining to the positive influence and effectiveness of technology, and increased interest in learning. Results were significantly higher in 3 of the 4 classes in the areas of increased interactivity, collaboration, and preference for a space to learn.

A final study from Byers and Imms (Imms and Byers, 2017) studied three classes (n = 170) of year 7 students in three different learning spaces: A traditional space (referred to as “mode 1”), a student-focused polycentric ALC (“mode 2”), and a “mode 3” learning space “where social activities overlap informal and active learning activities in spaces such as covered outdoor learning areas, hallway ‘nooks’ and lounge-styled rooms” (p. 140). Each class rotated through all three spaces, spending one academic term in each and taking the LTPS at the beginning and end of each term. The study found mixed results. On the LTPS items pertaining to student engagement (“domain C″ in the language of the LTPS), significantly higher ratings were given for the mode 3 classroom compared to the mode 1 (traditional) space, but not for the mode 2 space; significantly higher ratings for students’ willingness to take on challenges were obtained for both mode 2 and mode 3 compared to the traditional mode 1 space; and ratings for students’ willingness to work beyond their limits of expertise were statistically significantly higher only for mode 3 among only the high-ability students.

To round out our discussion of studies targeting general notions of engagement, we look at four other studies:

• A study by Whiteside et al. (2010), one of several conducted at the University of Minnesota, involved a quasi-experimental design in which one section of an introductory biology course was taught in a traditional lecture space, while another was taught in an ALC designed roughly according to the SCALE-UP model (large round tables seating 9 students each, ubiquitous projection technology, accessibility to whiteboards, etc.). An observational analysis was conducted to record the frequency and type of faculty and student behaviors in each section. The study found that students reported significantly higher ratings for ALCs on several parameters associated with engagement. Students rated ALCs higher for contributions to their class engagement overall, the enrichment of their learning experiences, the flexibility of their learning, and the fit of the classroom to the course. Additionally, there were significant differences in how students rated the different spaces based on their geographic background (urban versus rural) and their classification (freshman/sophomore versus junior/senior).
• In another study done at the University of Minnesota (Cotner et al., 2013), again two sections of an introductory biology course participated in a quasi-experiment in which one section was conducted in an ALC and the other in a traditional space. Surveys on student perceptions of those spaces were given during the last week of class, personal data were collected from students, and observations were conducted on a randomly selected 50% of those students with a focus on their on-task behavior and specific other learning behaviors. The study found significantly higher levels of engagement (measured generally through the survey and observational data) in the ALC and found significant positive correlation between the use of ALCs and the use of group activities.
• In a study of social science and literature classes in a Korean university (Park and Choi, 2014), one group of classes was conducted in an ALC holding 30 students using five circular tables outfitted with computer docking stations and projectors. Another group was held in a traditional space with a row-column arrangement of desks. Both groups were given a survey on their satisfaction with the rooms in which they learned. Students in the traditional space showed a preference for seating locations, which the authors termed the “golden zone”, as well as a “shadow zone” that was less preferred, and it was observed that the two different zones led to different learning outcomes --- and one's position in the room was strongly dependent on arrival time and proximity to friends. On the other hand, students in the ALC found it to be preferable to traditional learning spaces and found a much more uniform student experience of having a sense of belonging in the class, a sense of fun, and a high level of concentration and class attendance.
• In a smaller-scale study, Rands and Gansemer-Topf (2017) conducted a focus group of four instructors and nine students who had taught or taken classes in an ALC. The participants were given semi-structured interviews on their interaction with others, the physical and technological attributes of the room, and their perceptions of their own motivation and self-regulation. The researchers found that the ALC design helped to create a community of learners and “erased the line” between students and instructors. Respondents also reported that the open space in the ALC affords more movement and interaction, helps students work at their optimal level of challenge, provides tools that are helpful for assessing understanding and for visualizing thinking, and encourages holistic and integrated learning.

Engagement and Constructivism: As a final remark on these general results on engagement, we note one study (Sawers et al., 2016) that studied how instructors perceive “engagement” in students in an ALC. Instructors teaching in an ALC were given the Classroom Utilization Survey augmented with open-ended questions. The study found that the ALC tends to have a greater impact on perceived student engagement when used by faculty who identity with a more constructivist educational philosophy (that is, a teaching philosophy based on the notion that knowledge is not transferred from one person to another but rather constructed within the minds of each person through purposeful activity). It is unclear whether this means that actual student engagement is more frequent or pronounced in an ALC under a constructivist teacher, or whether more teachers subscribing to constructivism are more likely to interpret student activity as active engagement than those who are not constructivist. The results of Sawers et al. (2016) primarily serve as a caveat to those seeking to apply or interpret the research we have cited here, that survey and self-reported data must take into account the ontological stance of the observer.

#### 3.2.2 Specific categorical results about engagement

We also identified several studies that targeted specific forms of engagement. As we have noted, the categories in our engagement model strongly overlap, so our categorization below is approximate.

The following studies focused on affective forms of engagement:

• In Miller-Cochran and Gierdowski (2013), 195 students in sections of a university first-year writing course learned in an ALC using a “bring your own technology” (BYOT) approach to technology (in which students brought their own computing equipment, rather than it being provided as part of the space). The ALC in turn was outfitted with LCD projectors, whiteboards, and three kinds of moveable desks. In a post-semester survey on student preferences, 78% of students preferred the flexible BYOT design over traditional fixed design; 5% preferred more fixed design, and 17% indicated no preference. A large portion (66%) of students said the design of the ALC "somewhat contributes", "contributes", or "contributes highly” to their learning.
• In Hyun, Ediger, and Lee (2017), sixteen classes taught by seven different faculty were given an assessment focusing on student satisfaction and the frequency of engagement in active learning tasks. Five of the classes were taught in ALCs and the remaining eleven taught in traditional spaces. The study found that active learning pedagogy and classroom type were significantly correlated with levels of individual and group satisfaction and were significant predictors of both kinds of satisfaction. Student individual and group satisfaction were significantly increased in ALCs compared to corresponding measures of satisfaction in traditional classrooms.

The following studies focused on behavioral forms of engagement:

• Taylor (2009) examined two science classes taught in the same semester using a “Studio” setup (mentioned earlier as a precursor to the SCALE-UP idea). One class was an undergraduate introductory course, the other a graduate class. Each faculty member was interviewed four times and each class surveyed four times. The student surveys focused on overall impressions and usage of the Studio environment. The survey responses indicated that students attributed a significant impact on their learning to the ALC layout, particularly its affordances for group work and collaboration and for freedom of movement. Students also reported feeling more comfortable in asking questions of the instructor and of each other.
• In Whiteside, Jorn, Duin, and Fitzgerald (2009), two science classrooms (one engineering, the other biology) were renovated into ALCs using a variant of the SCALE-UP model. Surveys and exit interviews were conducted with faculty and students using the spaces (n = 17 and n = 168 respectively) along with 29 class observations focused on student attitudes and perceptions. Among other results reported in Section 6 (instructor practices), students expressed a broadly favorable opinion of the ALCs, especially in terms of their effectiveness to facilitate collaboration, connectedness, and discussion.
• A somewhat unusual study by Henshaw et al. (2011) involved taking a traditional classroom and replacing all the seats with swivel chairs, bolted to the floor but capable of 360-degree rotation. Through surveys given to students about their experiences in the room, the study found significant increases in face-to-face interaction among students and changes in instructor pedagogies that promote interaction (which we will describe in more detail in the next section).
• Another study (Ge et al., 2015) examined five classes with 92 students in all, along with five professors, in a “technology-enhanced” ALC. Each class was studied using classroom observations, interviews, and surveys at the beginning and end of the semester. By triangulating the various data, the researchers found that students achieved significantly higher confidence by the end of the semester, and students’ self-efficacy and confidence in problem solving tasks increased over time. However, there was no significant difference between the pre- and post-measures in terms of intrinsic motivation to solve problems. .
• Finally, Connolly and Lampe (2016) studied a class in information technology management being taught in an ALC with moveable furniture, multiple projector units, and a polycentric layout. Students reported that the classroom environment encouraged them to connect with fellow students, be more sociable, better prepare for class discussions, and enjoy the class more. However, we must also note that the survey data used in this study were not disclosed in the paper, and only three self-reported results from the surveys were mentioned.

Finally, the following studies focused on cognitive forms of engagement:

• Pashak and Hagen (2014) studied a single undergraduate psychology course with 24 students taught in a Steelcase LearnLab environment, with a polycentric layout featuring four tables seating six students each, and each table having a separate digital display switchable between different students at the table. The room also had PolyVision interactive whiteboards, personal whiteboards, and a document camera. Research assistants observed class sessions and noted behaviors of students including body movement and note-taking, as well as behaviors and pedagogical choices of the professors. Additionally, a survey was given to students at the end of the course to measure perceptions and preferences regarding the space. The main result of the study pertained to student attention: The timing of the class had a significant effect on students’ attention, and the study found lower levels of attention when the instructor was lecturing or conducting question-answer sessions, but higher levels of attention with less variation when engaged in question-and-answer with supervision. Additionally, the use of personal whiteboards was highly correlated with student attention.
• An extensive multiple case study of 232 students and 13 instructors in newly-constructed ALCs in a Canadian university (Gebre et al., 2015) found that students perceived that 43% of students whose professors held a “transmissive” view of effective teaching (i.e. primarily focused on direct instruction) felt their learning would have been the same or better in a traditional classroom, compared to just 27% and 8% of students whose professors viewed effective teaching as “engaging students” or “development” respectively.
• Stover and Zisweiler (2017) conducted a study of 417 students enrolled in “large lecture” courses (more than 70 students) at a university. In the Spring 2015 semester, the classes were taught in a traditional auditorium with fixed seating and primarily lecture pedagogy; in the following Fall semester, the class was taught in an ALC by faculty trained to use the affordances of the ALC, although the pedagogical practices of each faculty member were different. A survey was given to students in the final week of the semester consisting of 34 items from the Community of Inquiry survey. The study found that the ALC sections had a significantly *negative* effect on the perceived level of “instructor presence” in the course for one evening section of one professor and in all the sections of another professor. The ALC, on the other hand, had significantly positive impact on social presence in one remaining class. No statistically significant differences were found in any of the classes with regards to “cognitive presence”. We note that this is the only study in the review that reports back significantly negative results for ALC's compared to traditional spaces; Stover and Zisweiler speculate that this could be a function of the instructors’ pedagogical choices rather than the ALC itself.

### 3.3 What effect do ALCs have on the pedagogical practices and behaviors of instructors?

In this section we address the research question, *What effects do ALCs have on the pedagogical practices and behaviors of instructors?* The set of results that focus on instructors is considerably smaller than the set of results focusing on students; indeed, as we will mention later, the effects of ALCs on instructors is an emerging area for future research. Because of the smaller number of results on instructors, both practices and behaviors will be examined in this section. However, the data emerging from our review point to pedagogical practices and instructor behaviors as distinct dimensions of the impact of ALCs, so we will treat the results in separate subsections.

We only consider studies in which data were collected after an intervention involving a reasonably large group of instructors teaching in an ALC. Some studies that we found and which were treated one or more of the other research questions (for example, Chen (2014) and Connolly and Lampe (2016)) contain discussions about ALCs and instructors, but are excluded for this question, either because the sample size was too small (e.g. just one instructor teaching in the ALC was considered) or because the discussion is too general. Of the 37 studies in this review, we found 15 that address this particular question.

#### 3.3.1 Instructor pedagogical practices

Brooks (2012) conducted perhaps the most thorough study of the impact of ALCs on instructor pedagogical practices in this review. In his study, a custom classroom observation instrument was developed and used to observe the classes on 32 separate variables related to student and instructor activities, and administered to two sections of a course, one of which met in a traditional classroom space and the other in an ALC. The observations found significantly less lecturing done in the ALC (54.5% of observations compared with 77.4%), significantly more class discussion (48% more in the ALC), significantly less time spent at the podium (69.2% of observations versus 95.1%), significantly more time using marker boards (10.1% more frequently), and a higher rate of consulting with students in small groups (59.4% of observations versus 27.4%). There was no significant difference in the frequency of group activities or question-and-answer sessions in the two sections, or in the frequency of the use of Power Point slides. This can be explained by the fact that the instructor designed both sections with active learning in mind; and yet, even though the pedagogy was not planned to be different between the two sections, the differences in lecture and class discussion appeared nonetheless. In other words, in Brooks’ study, the ALC seemed to exert an influence on instructor pedagogy, causing the instructor to engage in more active pedagogical methods even when she was specifically told not to change instructional methods between sections.

Pashak and Hagen (2014) examined both student and instructor behaviors in a Steelcase LearnLab classroom, equipped with moveable tables and chairs, interactive whiteboards, huddle boards, and a document camera. They observed classes taught in the LearnLab and recorded the frequency of instructor behavior in five categories of actions: stationary lecture only, stationary question-and-answer, stationary supervision of student work, lecturing plus movement, and question-and-answer or supervision plus movement. They also measured the frequency of usage of five categories of technological tools: one PowerPoint slide for an extended period, a series of PowerPoint slides, huddle boards, a combination of tools, and no tools. The observations were done for a single course meeting in the LearnLab; i.e. there was no control group. Pashak and Hagan found significant differences among the five behavioral categories as well as among the five tool categories. Although “lecture” was still the most commonly used pedagogical method and “none” the most common tool category, further analysis revealed that the “question-and-answer with movement” format and the huddle board tool category were strongly correlated with student attention levels.

Metzger (2015) studied the use of team-teaching in an ALC. While the impact of the ALC on the pedagogical choices of the instructors was minimally reported, Metzger does conclude based on student data that team-teaching instruction in an ALC would be improved by setting up “zones of engagement” in the ALC, with different parts of the ALC used for different learning experiences and individual instructors on the team responsible for different zones. The flexibility of an ALC affords the opportunity to make such pedagogical choices.

Other studies investigated the impact of ALCs on instructor activities that were not strictly pedagogical choices but which impacted instruction. In Henshaw et al. (2011) a classroom was outfitted with seating attached to the floor but on a swivel that allowed 360 degrees of movement by students, along with large aisles to allow instructors to access students. Observational and interview data indicate that instructors in this room moved through the room more freely, which in turn created a positive impact on collaboration and group activity. In Rands and Gansemer-Topf (2017), a traditional classroom was renovated to include chairs and tables on casters, arranged in clusters seating four students each. As with the swivel chair configuration above, instructors in this ALC report that they moved around the classroom and engaged in discussions more frequently with students than they did in a traditional space.

#### 3.3.2 Instructor motivation and behavior

Whiteside et al. (2009) examined the “PAIR-up” model (“PAIR” being an acronym for Partner, Assess, Integrate, Revisit) and how faculty attitudes and practices change within an ALC in which this model is used. Through interviews and surveys of instructors, the researchers studied instructor expectations and attitudes before teaching in the ALCs and how those beliefs changed during the term. The faculty reported that the ALC changed or deepened student-teacher relationships, influenced the faculty to shift their role to more of a learning coach or facilitator, created an environment where learning could easily occur, and helped to minimize class preparation and allowed for more focus on content due to the ALC being already set up for active learning.

In a case study of five classes with 92 students and nine instructors in an ALC, Ge et al. (2015) report several influences on their motivations and behaviors about teaching directly attributable to the ALC. The professors reported a realization that they could move about the room and meet with students in an ALC, a change from their beliefs about teaching in a traditional classroom. They also reported that the ALC provided them enhanced opportunities to discuss students’ difficulties with the students, and that the technology afforded by the space helped them to visualize difficult concepts more easily.

Finally, Sawers et al. (2016) examined thirty instructors who taught in both a traditional space and an ALC to examine the effects of the instructors’ personal teaching philosophies on their instruction and its results. They found that instructors who adhere to a constructivist teaching philosophy perceived their students as being more engaged in an ALC than those instructors whose teaching philosophy was less constructivist; the data indicated no significant difference among teaching philosophies on the perception of student engagement in a traditional space. Further, the type of learning space used appeared to exert an influence on the relationship between instructor teaching philosophy, the use of active learning pedagogies, and perceptions of student engagement. The results of this study can be taken as a cautionary tale when interpreting the results of other studies involving self-reported data from instructors, namely that what instructors perceive in student activity can in large part be a function of their own pre-existing beliefs about learning.

### 3.4 What specific design elements of ALCs contribute significantly to other results?

Our fourth and final research question is, *What specific design elements of ALCs seem to make the biggest impact in the results on student learning, instructor practices, and engagement?* This question is important especially for school leaders and others making decisions about the construction of new ALCs or renovations of existing classroom spaces. The answers to this question may be particularly helpful for those seeking to make incremental transitions from traditional learning spaces to an ALC and are looking for a “minimum viable product” that will provide some of the benefits of an ALC for minimal investments in construction or redesign.

We note at the beginning that the studies in this review do not point to a single architecture or set of physical objects that drive the results in those studies. What does emerge from a close reading of the literature is a single design concept that is a common thread among the most pronounced positive results on ALCs: The concept of *connectedness*. Any architectural design, furniture, or tools included in an ALC that promote connectedness in any of several forms tends to drive the strongest results in the literature we have reviewed. We will now look at the major facets of connectedness as found in the studies we reviewed. Of the 37 studies in this review, we found 15 that address this particular question.

#### 3.4.1 Connectedness through mobility

Many of the results we have reviewed feature the freedom of movement as a key element in the positive results they obtained. In addition to the studies we have already reviewed, the following have a particular focus on the effects of mobility in the ALC.

Harvey and Kenyon (2013) focused on design attributes while comparing different types of ALC layouts. The study administered the Classroom Seating Rating Scale for Students (CSRS-S), comprised of 15 Likert-type items on subscales for “Comfort and Spaces”, “Learning Engagement”, and “Interactivity”. In their study, 863 students completed the CSRS-S rating five different kinds of seating: a modern mobile “Node” chair manufactured by Steelcase (which has a flexible attached desk and casters for mobility), stationary chairs with tables, fixed chairs with tables, chairs and tables without wheels, and stationary trapezoidal tables with chairs with wheels. The modern mobile chairs and the trapezoidal tables with chairs on wheels had the highest overall ratings, as well as the highest ratings for the three separate subscales, and those ratings were statistically significantly higher than the ratings for the other three forms of furniture. That is, the furnishings that enabled mobility the most were the ones most highly rated for the key aspects of the student experience in the classroom.

We have already mentioned a unique setting arrangement in a classroom studied by Henshaw et al. (2011) where the seats were bolted to the floor in four groups and allows for 360° rotation. The option for the instructors to move around including to the center of the space and the ability for the students to connects with their peers all around them had a strongly positive, impact on collaboration with peers and the instructor due to the ability to quickly and easily switch learning modes in the classroom and allow for free movement through the seats.

#### 3.4.2 Connectedness through visual relationships

Many of the results pertaining to SCALE-UP, as well as variants such as TEAL and PAIR-UP, note that the visual layout of the ALC makes a significant difference in how students and instructors perceive the purpose of the room as well as their own role in the space. The polycentric layout of these spaces, as we have already noted, directs the attention of students not to the front of the room occupied by an authority figure but to each other, fostering group cohesion and better enabling active learning.

A study by Salter et al. (2013) explored the effect of a unique collaborative design layout on collaboration as well as the use of technology. An entire building of collaborative spaces was constructed on their campus, each space of which being designed with different nonstandard shapes and layouts.

These layouts create **vertical zones** to facilitated separate types of activities, where groups in one level were able to work on completely different activities to groups working in other levels. In sequence, the highest level was the most desirable spot and encouraged students to join the class early, while the less desirable spot where at the entrance level of the class. Technology was incorporated as a supporting tool for every activity, from smaller team presentations to the entire class presentation. The collaborative learning space had high ratings from students in supporting collaboration and the use of the available technology. The most useable technologies were the big screen for the entire space, the small screens attached to tables, and laptop provided at the table. However, paradoxically, only half of the students in the study wished to take more courses in a collaborative space. The authors argue that this finding speaks to the need to help students orient themselves to the new learning expectations inherent in an ALC.

The work of Park and Choi (2014) found that traditional spaces with row-by-column seating tend to create zones, some of which are beneficial for learning while others are detrimental to learning, largely due to the front-centered visual layout of the room. To equalize the zones, the ALC was designed with wheels on the lectern, tables, and chairs to be able to reconfigure the layout easily. Students found the new space better for sight lines to the screens, in motivation to learn, in building relationships with other students, and other key facets of connectedness.

According to Taylor (2009), studio classroom spaces are characterized by a combination of moveable furniture that group students into learning teams, a centrally located or moveable teacher's station that does not create a “front” of the room, wireless laptops and computer projection, and wall spaces for writing or posting ideas. The goal is to create flexible spaces to support flexible pedagogy,hand-on activities. High flexibility and mobility have the opportunity for unfamiliarity, and as we mentioned in question four, greatest learning occurs in unfamiliar settings. Studio spaces are still unfamiliar layouts for many students, and the flexibility of the space means that it can be frequently reconfigured to create new learning settings. The study found that studio spaces were preferred by students due to their physical comfort and a less restrictive atmosphere.

Finally, we mention again the various studies of Terry Byers, Wesley Imms, and their collaborators (Byers et al., 2016; Byers et al., 2014; Byers and Imms, 2014, 2016; Imms and Byers, 2017), all of which involve comparing traditional spaces with “next generation learning spaces” with **polycentric layouts** capable of housing three different learning modes in the same space. All of those studies report back significantly higher ratings for ALCs compared to traditional spaces on measures of student engagement and learning outcomes. We specifically highlight the study by Imms and Byers (2017), in which three types of classes were evaluated: a traditional setting, a polycentric ALC that facilitates student-centered learning, and a space representing an informal setting which was referred to the “third space”. The study found a nuanced relationship between the three different modes; however, students generally preferred the third space (mode 3) over both of the other modes for fostering a positive attitude, taking on challenges, and working beyond the limits of their expertise.

#### 3.4.3 Connectedness through tools for learning

The ready availability of both analog and digital learning tools in an ALC promotes student activity that connects concepts to students’ own interests, experiences, and understanding; such activity also promotes the connection of knowledge by using the tools as sense-making implements.

Analog tools such as whiteboards are among the most-cited implements in an ALC that promote student learning and engagement. Beichner et al. (2007) describe the whiteboards as a “public thinking space” in the SCALE-UP class, where sharing knowledge and ideas is conducted. For content delivery modes, Brooks (2012) found significant difference in use of marker boards with ALC having higher frequency. In addition to wall-mounted whiteboards, personal whiteboards or “huddle boards” were one more source of communication which created special attention from students as reported by Pashak and Hagan (2014). Writable glass table tops were mentioned in Connolly and Lampe (2016) as another way of communicating in the ALC.

Digital tools also play a role in student sense-making activities. The several studies on SCALE-UP and related spaces focus in on the ready availability of digital tools such as digital projectors for student use, power supplies, and computers (either housed in the ALC or allowed through a “bring-your-own-technology” approach) as key elements of improved student learning and interaction in the classroom. Conversely, Byers and Imms (2016) investigated the interaction between learning space and digital technology, and how this interaction affects teaching and learning in primary schools and found that students in an ALC perceive the technology as being more beneficial for their learning than the same technology used in the same ways in a traditional space. As Connolly and Lampe (2016) also point out, the availability of digital projects makes it easier to have a room with a polycentric layout, since student visual attention can be focused in a variety of directions when a digital item is on display.

## 4 Discussion

In this section, we will summarize the primary themes of the findings in the studies identified for this review. We also discuss the limitations and methodological issues in those studies.

### 4.1 Primary themes

Looking across all the research questions from studies in this review, the following common themes emerge from research on ALCs.

*ALCs are connected with improved student learning outcomes*. ALCs tend to be associated with improved measurable student learning outcomes, whether those outcomes are traditional quantitative measures such as exams and course grades or measures of "21st century skills". All of the studies in the review that reported on measurable student learning outcomes reported either improved outcomes for students in ALC sections, or no significant difference in learning outcomes when comparing ALC sections to sections in traditional classroom spaces. None reported lower results for students in ALC sections. Furthermore, several of the studies report that the results on learning outcomes are the most pronounced among low-achieving and minority students.

*ALCs are connected with improved student engagement, in several forms.* ALCs tend to provoke strong improvements in student engagement, framed in terms of affective, behavioral, or cognitive forms or as a combination of these. Students typically report a preference for learning in an ALC compared to a traditional space as well as increased motivation to attend class. Students also report increased willingness to participate actively in class and to take on challenges and work past their comfort zone in an ALC versus in a traditional space. Students also report that ALCs lead to increased interaction and deepened relationships with their peers and instructors, and that ALCs foster a sense of community and belonging.

*ALCs have a positive connection with instructor practices and beliefs.* When focusing from students to instructors, a third theme emerges: Instructors in ALCs tend to change their instructional practices and their perceptions of their role as instructors. Our studies report that instructors in ALCs tend to use active learning techniques more frequently than they do when teaching in traditional classrooms, and they readily integrate the special affordances of ALCs --- reconfigurable tables, vertical writing surfaces, ubiquitous digital technology, etc. --- into their teaching effectively. One study (Brooks and Solheim (2014)) reported increases in the use of active learning and ALC affordances even when instructors were specifically told to teach the same way they do in a traditional lecture environment. Several of the studies also report that the experience of teaching in an ALC can drive fundamental reconsiderations of instructors’ teaching goals and philosophies. However, other studies indicate that those changes in mindset are to a significant extent dependent on the starting point of instructors’ teaching philosophies.

*ALCs are a potential tool for the evolutio*n *of a new culture of learning.* A fourth theme emerges from the literature as a combination of the above three themes, namely that there is a significant potential for ALCs to have a transformative effect on the institutional and learning cultures of the schools in which they are installed. By "institutional culture", we mean the norms, practices, and beliefs of an educational institution that shape the way it and its members perform their work and interact with each other; by "learning culture", we similarly mean the norms, practices, and beliefs held and enacted by instructors and learners as it pertains to learning. While none of the studies in this review specifically studied the institutional or learning cultures of schools, for example using anthropological methods, there appears to be evidence that a synergy between active learning and ALCs has a significant, system-wide impact on the social and cultural patterns in the schools where ALCs are found and in which active learning is used.

For example, the results we have summarized pertaining to student engagement all point to greatly enhanced engagement, however it may be defined, in the presence of ALCs and the active learning that take place within those spaces. The work of Scott-Webber et al., 2014), in particular, indicates that students learning in an ALC rate ALCs significantly engaging on all 12 levels testing by the instrument used in that study, including "feeling comfortable to participate” and "creation of an enriching experience". Many of the studies in the review also report repeated instances of students finding learning in an ALC to be more “fun” than in a traditional space; whether this refers to the space, or to pedagogy or technology, is unclear, but the perception that learning is fun represents a significant cultural shift for most students.

Similarly, the various studies by Byers and Imms and their collaborators show that learning in an ALC leads to enhanced experiences in general areas of student engagement and student learning experiences. Some of the specific forms of engagement and learning experience pertain to broader, big-picture aspects of the student experience such as comfort level, the willingness to work outside of one's comfort zone, and perceptions of what teaching effective. These larger systemic items in these and other studies point to the beginnings, at least, of major changes in the ways that students conceive of their own roles as learners in a school, and what learning looks and feels like. These are at the root of large-scale cultural change.

The results for instructors point primarily to the fact that the presence of an ALC tends to encourage instructors to use more active learning techniques in class, and this in turn often causes instructors to rethink their own roles in their profession. For example, Brooks and Solheim (2014) indicates that instructors use more active learning in an ALC even when specifically instructed not to do so; and faculty, after these experiences, tend to view themselves more as a guide or coach than as a lecturer.

The common denominator in the larger cultural effects of ALCs and active learning on students and instructors is the notion of *connectedness,* a concept we have already introduced in discussions of specific ALC design elements. By being freer to move and have physical and visual contact with each other in a class meeting, students **feel more connected to each other and more connected to their instructor**. By having an architectural design that facilitates not only movement but choice and agency --- for example, through the use of polycentric layouts and reconfigurable furniture --- **the line between instructor and students is erased**, turning the ALC into a vessel in which an authentic community of learners can take form. By having analog and digital tools readily available in the ALC, students are better able to connect the concepts of a class to their own interests and conceptions and are better able to draw connections between ideas. A learning experience promoting a significantly enhanced level of connectedness on a number of levels would, by itself, indicate a major cultural shift in current educational spaces and practices. While, again, none of the results in this study specifically focus on cultural change, this aspect of the presence and effect of the ALC indicates that cultural change is there to be discovered with future research.

### 4.2 Limitations and issues in the studies and their results

As with any research study, the studies in our review had limitations in their design and methodology that should be kept in mind when interpreting and applying their results.

*Failure to control for pedagogy and technology*. The first and by far most prevalent methodological limitation is that many of the studies in this review do not control for variables related to pedagogy or technology. For example, the various SCALE-UP studies appearing in this review often report significant improvements in student learning outcomes and behaviors. But it is unclear from the studies what, precisely, is primarily responsible for these results: the space itself, the active learning taking place within the space (and whether similar results might be obtained by similar pedagogy in a more traditional space), the availability and use of technology, or some multiple-way interaction between these. Therefore, it is difficult or impossible to distinguish between the effects of the learning space on the one hand, and the effects of the pedagogy and technology used in that space on the other, in most of the studies in this review. Many of the studies report instructor and student beliefs that ALCs at least afford greater opportunities for engaging in effective active learning and technology integration. But in terms of isolating the role of space by itself and controlling for the variables of pedagogy and technology, only the studies involving Terry Byers and Wesley Imms explicitly design their research to accomplish this, and research that delineates between space, pedagogy, and technology is still emerging.

*Limited cultural and educational contexts*. Second, as the overview of the results in Section 2 show, the cultural and educational contexts of these studies are quite limited. The vast majority of the studies in the review were done in higher educational settings, with only five being conducted in primary or secondary schools. We find it unusual, give the tremendous amount of interest in primary and secondary schools in ALCs, that the body of published research on ALCs in this setting is so small. Also, a large majority of the studies (27 out of 37) in this review were done in the United States; half of the remaining 10 studies were conducted in Australia. The combination of the limitations on educational and cultural contexts make many of the results difficult to generalize outside of their niche contexts, and those wishing to apply the results from this review to contexts outside these contexts should take special care in generalizing.

*Use of quantitative measures that lack standardization and rigor*. Third, the studies in the review that focus on quantitative indicators of learning, tend to use measures such as exam grades and course grades, using instruments that are typically designed by individual faculty members and not subjected to rigorous analysis of their reliability and thus have uncertain internal validity. For example, course grades often include group work or attendance credit which do not correlate with individual student learning outcomes; and exams given to courses with different instructors are not always examined for inter-rater reliability. The studies that use normalized learning gains on standardized concept inventories have considerably stronger validity but are fewer in number.

*Limited scope and sample size*. Fourth, several of the studies use limited samples. Some studies identified in the database searches involved even more limited samples, for example just three responses to a survey; those studies were excluded from review. Many of the remainder that passed our inclusion criteria were still quite limited in their scope, often to just to sections of a single course for a single instructor in one semester. As with cultural and educational context limitations, the limited sampling scope makes it difficult to generalize the results of those studies with confidence.

## 5 Conclusion

Our understanding of the role that learning spaces play in the learning process is still in its adolescence, but the research we have reviewed here, along with the numerous new studies published since the writing of this manuscript, speak to the growing importance of this role in all phases of education. The research emerging from the study of learning spaces highlights a growing need to understand space as a third component of effective learning experiences, complementing pedagogy and technology:

As our understanding of both pedagogy (particularly active learning pedagogies) and technology (particularly technology used for active learning tasks) evolves, a continuing evolution of our understanding of active learning spaces is necessary to help the promise of active learning reach its full potential.

To conclude this review, we will identify emerging lines of inquiry about active learning spaces that hold promise for future research. Many of these opportunities for future research are based on the limitations of the research that we have reviewed in this study.

*Research outside higher education contexts*. As noted, a great majority of the research reviewed here is done in higher education. This research may not be particularly useful for primary and secondary schools, given the wide differences in organizational and educational norms between primary/secondary and higher education. In fact, a great deal of work on ALCs that is either unpublished or non-peer reviewed exists in the primary and secondary education space in the form of editorials, blog posts, action reports from classroom usage, conference presentations, and reports from grants such as Steelcase, Inc.‘s Active Learning Center Grant program. Given the strong interest in active learning classrooms from primary and secondary schools and the relative lack of published research done in those contexts, more research done in these schools is warranted. One avenue for generating such research would be to build on the extensive body of existing work that is unpublished or non-peer reviewed, and expand those works into research studies of the kind we have reviewed here.

*Research in a wider variety of ethnic and cultural contexts*. Similarly, there is a lack of research on ALCs situated in countries outside the United States. Even within the US studies, all of the studies were done in universities, the plurality of which are large research-focused institutions. Research that investigates ALCs in a wider diversity of cultural context would be useful for teasing apart the role of culture and ethnicity in the effects we have documented here.

*Research that controls for pedagogy and technology*. As we noted in the previous section, most of the studies reviewed here do not attempt to control for pedagogical methods or technology use. As a result, it remains unclear in many cases whether the results we have seen in this review can be isolated and attributed to the space itself, versus the active pedagogy and intentional use of technology (or combination of these) taking place in the space. Terry Byers and Wesley Imms, along with their collaborators, have specifically targeted methodologies that do control for these variables, and consequently their results reflect the influence of space alone. More such research is needed to determine which results depend on the particular ALC space involved. We believe that replications of existing studies with appropriately-adjusted research methodology, for example replications of SCALE-UP studies using single subject research designs, would represent an important step in this direction.

*Longitudinal research*. We did not encounter any longitudinal studies in this review. As more ALCs are constructed around the world in schools and universities, the opportunities to conduct long-term studies of the persistence of ALCs’ effectiveness grow proportionately. The time seems right for longitudinal research, which would extend the results found in the studies in this review over time and through different kinds of spaces to see if the results especially regarding changes in attitude and behavior persist.

*Research comparing different models of ALCs*. Only one of the studies in this review (Muthyala and Wei, 2013)compared two different layouts of an ALC for their relative effects on learning outcomes. (There were no significant differences in those outcomes between the two layouts.) This seems to be a promising entry point for research, particularly for schools that have already installed ALCs and can simply reconfigure them to create different groups for study while controlling for confounding variables.

*Research on design and architectural principles that impact learning*. Our final research question (*What specific design or architectural elements of ALCs contribute the most to the results in the identified studies?*) was largely answered deductively by looking at patterns in the research data. None of the studies we encountered specifically aimed to address this question. Studies that examine design and architectural elements, while keeping other variables under control, would be useful for designers, architects, and potential buyers of ALCs.

*Ethnographic research on cultural effects*. In the previous section, we noted the strong potential for cultural change that is attached to the use of ALCs; however, we also noted that none of the studies in this review directly address cultural change in institutions adopting ALCs. We believe the issue of systemic culture change merits further, more targeted research. Specifically, ethnographic research done within institutions that adopt ALCs to examine the cultural impact of "connectedness” as discussed in the previous section, collaboration, territorial behaviors, and other instances of culture could yield significant insight on the impact of ALCs not directly tied to student learning.

## Declarations

### Author contribution statement

All authors listed have significantly contributed to the development and the writing of this article.

### Funding statement

This work was commissioned by 10.13039/100004685Steelcase, Inc. as a project for the sabbatical leave of one of the authors (Talbert), and Steelcase provided work space and partial funding of that author's salary during the sabbatical. Steelcase provided funding for the participation of the other author (Mor-Avi).

### Competing interest statement

The authors declare the following conflict of interests: This work was commissioned by Steelcase, Inc. as a project for the sabbatical leave of one of the authors (Talbert), and Steelcase provided work space and partial funding of that author's salary during the sabbatical. Steelcase provided funding for the participation of the other author (Mor-Avi).

### Additional information

No additional information is available for this paper.

## Footnotes

1 We wish to note that our use of the term “active learning center” is not connected to the “Active Learning Center Grant” program offered by Steelcase, Inc. but is merely the most common term used to describe these spaces.

## References

[bib1] Ananiadou K., Claro M. (2009). Working Paper 21st Century Skills and Competences for New Millenium Learners in OECD Countries. Edu/Wkp.

[bib2] Astin A. (1999). Student involvement: a developmental theory for higher education. J. Coll. Student Dev..

[bib3] Axelson R.D., Flick A. (2010). Defining student engagement. Change.

[bib4] Baepler P., Walker J.D., Brooks D.C., Saichaie K., Petersen C. (2016). A Guide to Teaching in the Active Learning Classroom.

[bib5] Baepler P., Walker J.D., Driessen M. (2014). It’s not about seat time: blending, flipping, and efficiency in active learning classrooms. Comput. Educ..

[bib6] Beichner R. (1999). Case study of the physics component of an integrated curriculum. Am. J. Phys..

[bib7] Beichner R.J., Saul J.M., Abbott D.S., Morse J.J., Deardorff D., Allain R.J., Risley J. (2007). The student-centered activities for large enrollment undergraduate programs (SCALE-UP) project abstract. Physics.

[bib8] Bonwell C., Eison J. (1991). Active Learning: Creating Excitement in the Classroom.

[bib9] Brooks C., Brown M., Buckner M., Cotner S., Finkelstein A. (2017). The Year of the Active Learning Classroom. http://bit.ly/2Ru2CcU2019-01-14.

[bib10] Brooks D.C. (2011). Space matters: the impact of formal learning environments on student learning. Br. J. Educ. Technol..

[bib11] Brooks D.C. (2012). Space and consequences: the impact of different formal learning spaces on instructor and student behavior. J. Learn. Spaces.

[bib12] Brooks D.C., Solheim C.A. (2014). Pedagogy matters, too: the impact of adapting teaching approaches to formal learning environments on student learning. New Directions for Teaching and Learning.

[bib13] Byers T., Hartnell-Young E., Imms W. (2016). Empirical evaluation of different classroom spaces on students’ perceptions of the use and effectiveness of 1-to-1 technology. Br. J. Educ. Technol..

[bib14] Byers T., Imms W. (2014). Making the space for space: the effect of the classroom layout on teacher and student usage and perception of one-to-one technology. Aust. Comp. Educ. Conf..

[bib15] Byers T., Imms W. (2016). Evaluating the change in space in a technology-enabled primary years setting. The Translational Design of Schools.

[bib16] Byers T., Imms W., Hartnell-Young E. (2014). Making the case for space: the effect of learning spaces on teaching and learning. Curric. Teach..

[bib17] Chen V. (2014). “There is No single right answer”: the potential for active learning classrooms to facilitate actively open-minded thinking. Collect. Essays Learn. Teach..

[bib18] Chiu P.H.P., Cheng S.H. (2016). Effects of active learning classrooms on student learning: a two-year empirical investigation on student perceptions and academic performance. High. Educ. Res. Dev..

[bib19] Coletta V.P., Phillips J.A. (2005). Interpreting FCI scores: normalized gain, preinstruction scores, and scientific reasoning ability. Am. J. Phys..

[bib20] Connolly A., Lampe M. (2016). How an active learning classroom transformed IT executive management. Inf. Syst. Electron. J..

[bib21] Cotner S., Loper J., Walker J.D., Brooks D.C. (2013). It’s not you, it’s the room - are the high-tech, active learning classrooms worth it?. J. Coll. Sci. Teach..

[bib22] Dori Y.J., Belcher J. (2005). How does technology-enabled active learning affect undergraduate students’ understanding of electromagnetism concepts?. J. Learn. Sci..

[bib23] Eagly A.H., Chaiken S., Gilbert D.T., Fiske S.T., Lindzey G. (1998). Attitude structure and function. The Handbook of Social Psychology.

[bib24] Framework for 21st Century Learning - P21 (2019). http://www.p21.org/about-us/p21-framework.

[bib25] Freeman S., Eddy S.L., McDonough M., Smith M.K., Okoroafor N., Jordt H., Wenderoth M.P. (2014). Active learning increases student performance in science, engineering, and mathematics. Proc. Natl. Acad. Sci..

[bib26] Ge X., Yang Y.J., Liao L., Wolfe E.G. (2015). Perceived affordances of a technology-enhanced active learning classroom in promoting collaborative problem solving. E-learning Systems, Environments and Approaches, (Celda).

[bib27] Gebre E., Saroyan A., Aulls M.W. (2015). Conceptions of effective teaching and perceived use of computer technologies in active learning classrooms. Int. J. Teach. and Learn. Higher Educ..

[bib28] Gierdowski D. (2013). Studying learning spaces: a review of selected empirical studies. Cases on Higher Education Spaces: Innovation.

[bib29] Hall E.T. (1959). The Silent Language.

[bib30] Halloun I.A., Hestenes D. (1985). The initial knowledge state of college physics students. Am. J. Phys..

[bib31] Harvey E.J., Kenyon M.C. (2013). Classroom seating considerations for 21st century students and faculty. J. Learn. Spaces.

[bib32] Henshaw R.G., Edwards P.M., Bagley E.J. (2011). Use of swivel desks and aisle space to promote interaction in mid-sized college classrooms. J. Learn. Spaces.

[bib33] Grajek Susan (2015). Higher Education’s Top 10 Strategic Technologies for 2017. https://library.educause.edu/resources/2017/1/higher-educations-top-10-strategic-technologies-for-2017.

[bib34] Hyun J., Ediger R., Lee D. (2017). Students’ satisfaction on their learning process in active learning and traditional classrooms. Int. J. Teach. and Learn. Higher Educ..

[bib35] Imms W., Byers T. (2017). Impact of classroom design on teacher pedagogy and student engagement and performance in mathematics. Learn. Environ. Res..

[bib36] Metzger K.J. (2015). Collaborative teaching practices in undergraduate active learning classrooms: a report of faculty team teaching models and student reflections from two biology courses. Bioscene.

[bib37] Meyers-Levy J., Zhu R., Juliet (2007). The influence of ceiling height: the effect of priming on the type of processing that people use. J. Consum. Res..

[bib38] Miller-Cochran S., Gierdowski D. (2013). Making peace with the rising costs of writing technologies: flexible classroom design as a sustainable solution. Comput. Compos..

[bib39] Muthyala R.S., Wei W. (2013). Does space matter? Impact of classroom space on student learning in an organic-first Curriculum. J. Chem. Educ..

[bib40] Nissim Y., Weissblueth E., Scott-Webber L., Amar S. (2016). The effect of a stimulating learning environment on pre-service teachers’ motivation and 21st century skills. J. Educ. Learn..

[bib41] OCDE (2009). Working paper 21st century skills and competences for new millenium learners in OECD countries. Edu/Wkp.

[bib42] Oliver-Hoyo M.T., Allen D., Hunt W.F., Hutson J., Pitts A. (2004). Effects of an active learning environment: teaching innovations at a research I institution. J. Chem. Educ..

[bib43] Park E.L., Choi B.K. (2014). Transformation of classroom spaces: traditional versus active learning classroom in colleges. High. Educ..

[bib44] Pashak T.J., Hagen J.W. (2014). The LearnLab : using enhanced teaching technology to improve learning in the college classroom. Curr. Adv. Psychol. Res..

[bib45] Rands M.L., Gansemer-Topf A.M. (2017). The room itself is active: how classroom design impacts student engagement. J. Learn. Spaces.

[bib46] Salter D., Thomson D.L., Fox B., Lam J. (2013). Use and evaluation of a technology-rich experimental collaborative classroom. High. Educ. Res. Dev..

[bib47] Sawers K.M., Wicks D., Mvududu N., Seeley L., Copeland R. (2016). What drives student engagement: is it learning space, instructor behavior, or teaching philosophy?. J. Learn. Spaces.

[bib48] Scott-Webber L., Abraham J., Marini M. (2000). Higher education classroom fail to meet needs of faculty and students. J. Inter. Des..

[bib49] Scott-Webber L., Konyndyk R., French R., Lembke J., Kinney T. (2017). Spatial design makes a difference in student academic engagement levels: a pilot study for grades 9-12. Eur. Sci. J..

[bib50] Scott-Webber L., Strickland A., Kapitula L. (2014). Built environments impact behaviors: results of an active-learning post-occupancy evaluation. Plan. High. Educ. J..

[bib51] Stanovich K.E., West R.F. (1997). Reasoning independently of prior belief and individual differences in actively open-minded thinking. J. Educ. Psychol..

[bib52] Stover S., Ziswiler K. (2017). Impact of active learning environments on community of inquiry. Int. J. Teach. and Learn. Higher Educ..

[bib53] Taylor S.S. (2009). Effects of studio space on teaching and learning: preliminary findings from two case studies. Innov. High. Educ..

[bib54] Van Horne S., Murniati C., Gaffney J.D.H., Jesse M. (2012). Case study: promoting active learning in technology-infused TILE classrooms at the university of Iowa. J. Learn. Spaces.

[bib55] Walker J.D., Brooks D.C., Baepler P. (2011). Pedagogy and space : empirical research on new learning environments. Educ. Q..

[bib56] Whiteside A.L., Brooks D.C., Walker J.D. (2010). Making the case for space: three years of empirical research on learning environments. Educ. Q..

[bib57] Whiteside A.L., Jorn L., Duin A.H., Fitzgerald S. (2009). Using the PAIR-up model to evaluate active learning spaces. Educ. Q..

[bib58] Wilson J.M. (1994). The CUPLE physics studio. Phys. Teach..

[bib59] Wilson J.M., Jennings W.C. (2000). Studio courses: how information technology is changing the way we teach, on campus and off. Proc. IEEE.

