# Supplementary material for: A space for learning: An analysis of research on active learning spaces
Source: Heliyon. 2019 Dec 24;5(12):e02967. doi: 10.1016/j.heliyon.2019.e02967 (PMC7190693; doi:10.1016/j.heliyon.2019.e02967)
Supplement: Appendix A [file mmc1.docx]

**Appendix A: Summary Table of Results**

**Note:** Under “Questions”, Q1 through Q4 refer to the four main research questions discussed in Section 2.1.

| **Authors** | **Year** | **Context** | **Location** | **Questions** | **Intervention** | **Size** | **Study Design** | **Results** |
| --- | --- | --- | --- | --- | --- | --- | --- | --- |
| Baepler, Walker, & Driessen | 2014 | Higher education | USA | Q1, Q2 | General chemistry class of 350 students meeting 3 times per week in traditional space split into three groups and met in 117-seat ALC, each cohort meeting once per week, with remaining days spent on online activities. | 350 | Quasi-experimental post-test only nonequivalent groups design. Data collection included grades, demographics, and survey. Two 20-item multiple choice exams designed by ACS and one 40-item exam designed by faculty administered. | Moderate performance difference on ACS exam between experimental group 1 and control; no significant difference between experimental group 2 and control. After controlling for demographic and aptitude variables, flipped/blended+ALC approach yielded results the same as or better than traditional classroom experience. |
| Beichner et al. | 2007 | Higher education | USA | Q1, Q2, Q4 | Introduction of SCALE-UP ALCs in large-section introductory physics courses. Study examines results comparing over 16000 students over five years in traditional vs. SCALE-UP classrooms. | Varies | Quasi-experimental: Students' learning gains are compared in traditional vs. SCALE-UP courses on the Force Concept Inventory, Conceptual Survey on Electricity and Magnetism, and Electrical Circuit Conceptual Evaluation as well as failure ratios and a variety of affective measures. | Conceptual understanding increased in SCALE-UP with the top third of classes showing the greatest improvement; ability to solve problems as good or better in SCALE-UP; attitudes and attendance are improved; failure rates are reduced in SCALE-UP typically by 50% with greatest improvements in failure rates among women and minorities; performance in second-semester physics is improved; failure of at-risk students in later Engineering Statics class was cut in half. |
| Brooks | 2011 | Higher education | USA | Q1 | Introductory biology course was taught in new ALCs with round tables seating 9 students each, with switchable laptop technology to feed student screens to large projectors, along with glass whiteboards around the room. | 83 | Quasi-experimental: Pre-post design with one section in ALC and the other in a traditional classroom. Only significant variable was space and ACT score with the traditional section having a higher ACT average. Instructional space was varied, and course grades measured as output. | Regression model predicted significantly lower course performance for the ALC section based on ACT scores, but the difference in course performance was not statistically significant; authors conclude that the ALC closed an achievement gap that would otherwise have been visible. |
| Brooks | 2012 | Higher education | USA | Q2, Q3 | Introductory biology classes, one section taught in a traditional class and the other taught in an ALC, were given a custom classroom observation instrument to measure 32 variables related to classroom activity, content delivery mode, instructor behavior, levels of on-task student behavior, environmental conditions, narrative descriptions of events. | 208/224 | Observational: Class observations made with the custom instrument in randomly-scheduled unannounced class periods in 46.4% and 50.0% of meetings traditional and ALC rooms respectively. Data were recorded in 5-minute intervals (total of 208 and 224 observations for traditional and ALC). | Significant difference in lecture frequency with traditional room having more lecture than ALC; no significant differences in frequency of group activity or Q&A sessions. Significant difference in use of marker boards with ALC higher; no significant difference in frequency of use of PowerPoint slides. Significant differences in use of lecture (traditional more frequent), consulting in small groups (ALC more frequent), and other group engagement (ALC more frequent). Significant difference in frequency of on-task student behavior at all but lowest level, with traditional students exhibiting more on-task behavior. |
| Brooks & Solheim | 2014 | Higher education | USA | Q1, Q2 | Personal finance course taught in an ALC. No change in space, but pedagogy was changed from lecture to team-based learning. Faculty development programming was provided for transition to TBL. | 111 | Quasi-experimental: Comparing differences in course grades and assignments between lecture and TBL sections, as well as quantitative survey assessment of student perceptions | Students in TBL section had higher course grades than students in lecture section (p < 0.001) and higher grades on individual major assignments (all at p < 0.001, some at p < 0.0001). Students in TBL section responded more strongly to survey questions about encouragement of active participation, otherwise no significant differences on survey results. |
| Byers & Imms | 2014 | Primary/secondary | Australia | Q2, Q4 | Three buildings constructed between 1940 and 1960; each building with six traditional classrooms. Rooms remodeled to provide three spatial modalities and a polycentric layout. | 164 | Single-subject design: Classes in English and Mathematics were taught in traditional class, then ALC. Students (n = 164) were given survey consisting of three groups of three questions each, measuring "Positive Influence", "Effectiveness", and "Flexibility" of one-to-one technology in the classroom. Two year-7 and four year-8 classes were involved. | Statistically significant differences were found in 12 out of 18 question groups with ALCs receiving higher ratings in positive influence, effectiveness, and flexibility. Only non-significant results were in positive influence and flexibility in one year-7 class and in two year-8 classes. Clear statistical differences found in five out of six classes with student learning experiences rated higher in the ALC than in the traditional class. Statistically significant differences on engagement in all six classes, with higher engagement reported in ALC. Teachers in focus group reported high levels of excitement and engagement in the ALC. |
| Byers & Imms | 2016 | Primary education | Australia | Q2, Q3, Q4 | Two classrooms involved: One traditional space, rows of desks (could be rearranged) facing front of room, interactive whiteboard present along with wireless internet and tablet computers. The other an ALC with adjustable-height tables and polycentric layout. Students involved (n = 94) were year 4 students in variety of academic subjects. | 94 | Single-subject design: All subjects and all facets of instruction kept constant except space -- students were moved from traditional space to ALC and then back. Students were given Linking Pedagogy Technology and Space (LPTS) survey (10 items, 3 domains: effect of learning spaces on students’ perceptions of technology, learning experiences, engagement). | Statistically significant differences on 9 out of 16 questions with higher/more favorable responses given for ALC. Positive influence and effectiveness of technology. Little effect on level of student distraction attributed to use of technology. Non-significant differences in teacher practice and personalization of instruction. Significant differences on interactivity and collaboration. Also significant differences on interest in learning and preferred place to learn. |
| Byers, Hartnell-Young & Imms | 2016 | Secondary education | Australia | Q2 | Three buildings at single school for years 7,8, and 9 with two ALCs: One with traditional row x column layout, the other with polycentric layout. Both had projectors, screens, wireless networking, and Tablet PC's. Study involved 385 students and 21 teachers from 22 classes in English, Humanities, and Mathematics. | 385 | Single-subject design: Classes divided into ALC intervention group (spending one semester in each of the two layouts), ALC control group (both semesters in ALC), and traditional control group (both semesters in traditional space). Groups given attitudinal surveys consisting of 5-point likert scale items on incidence in use of digital technology, effectiveness of digital technology, and effect of space on technology. A focus group of teachers was also held. | Spatial intervention did not significantly affect incidence of student use of technology. Significant effects of intervention found on effectiveness of technology use in five classes, two of which attributed to the timing of the intervention and remaining three due to latency effect. Statistically significant differences in perceptions of digital technology. ALC students had more favorable view of impact of space on the technology use vs. traditional space. |
| Byers, Imms & Hartnell-Young | 2014 | Primary/secondary | Australia | Q2 | Three buildings constructed between 1940 and 1960; each building with six traditional classrooms. Rooms remodeled to provide three spatial modalities and a polycentric layout. | 164 | Single-subject design: Classes in English and Mathematics were taught in traditional class, then ALC. Students (n = 164) were given survey consisting of three groups of three questions each, measuring "Positive Influence", "Effectiveness", and "Flexibility" of one-to-one technology in the classroom. Two year-7 and four year-8 classes were involved. | Statistically significant differences found in 12 out of 18 question groups with ALCs receiving higher survey ratings in positive influence, effectiveness, and flexibility. Only non-significant results were in positive influence and flexibility in one year-7 class and in two year-8 classes. Clear statistical differences found in five out of six classes with student learning experiences rated higher in the ALC than in the traditional class. Statistically significant differences on engagement in all six classes, with higher engagement reported in ALC. Teachers in focus group reported high levels of excitement and engagement in the ALC. |
| Chen | 2014 | Higher education | USA | Q2 | The participants were 28 4th year psychology students, and the course was designed with strong emphasis on group work. The procedure was on-line in Fluid Survey. Circled tables for 6 students with media screens around the room. | 28 | Actively Open-minded Thinking questioner (Stanovich and West's 2007)- AOT + conversations between the teacher and the students along the term. (Note: There was no control group) | Most of the students found learning in an ALC with the dynamic curriculum as a very good experience.  Students liked: 1. Interact with peers 2. Get ideas and learn to listen and even change views accordingly 3. Less competitive atmosphere According to the instructor point of view, this class was a big success and enable her to change the pedagogy of the course to a more dynamic and active. Space changed their perception of what could be achieved from the course which in turn had a better potential to influence on students’ open-mindedness. This study shows the potential of developing a better open-minded thinking during the semester while using ALC. This study shows the potential of developing a better open-minded thinking during the semester while using ALC. |
| Chiu & Cheng | 2016 | Higher education | Hong Kong | Q2 | 306 general education courses taught campus wide and across disciplines, some in ALCs and others in traditional spaces. ALC had max capacity of 50 students, seated at hexagonal tables. | 35953 | Survey: Students (n = 35953) over two-year period responded to Teaching and Learning Questionnaire (7-point likert scale questions giving general course and learning feedback). | Significantly better learning experiences in ALC for course design and encouragement to be creative and innovative; effect persists across academic years. ALC group in second year had significant differences in course difficulty — rated ALC courses as being less difficult. In-class learning experience and encouragement to be creative and innovative compared with academic performance in terms of GPA; ANOVA shows significant change in means for ALC group vs traditional group on “encouragement to be creative and innovative"; student perceptions of being creative/innovative were significantly better for ALC than for traditional but no interaction statistically between this effect and academic performance. |
| Connolly & Lampe | 2016 | Higher education | USA | Q2, Q4 | First class out of 8 class project to evaluate active learning. This class designed for IT management curricula. ALC used movable furniture, tables with writeable tops, multiple projectors, ad polycentric layout. | Not given | Students and faculty given surveys and focus groups at the end of the term. | Students reported that the classroom environment affected their learning. Students stated that the classroom arrangement encouraged them to get to know their fellow students, to come out of their shells, to network with classmates, and to better prepare for class discussions. Their critical thinking improved. Students and faculty enjoyed the course.They did not entirely trust their control over the space |
| Cotner, Loper, Walker, & Brooks | 2013 | Higher education | USA | Q1, Q2, Q3 | Introductory biology course for non-biology majors, one instructor and two sections, one section in an ALC and the other in a traditional space. (n = 161 and 102 respectively) | 161/102 | Quasi-experiment. Surveys on their perceptions of their spaces given during last week of class; personal data collected from students; observations done on randomly selected 50% of students and recorded levels at which students were on task, as well as specific learning behaviors being exhibited; final grades in each section recorded. | Students in ALC performed significantly better on course grades (half letter grade higher) than their initial ACT scores predicted. ALC instructors spent more time consulting and leading group activities and less time at podium than instructors in traditional space. Significant positive correlations between the use of ALCs and group activities. ALC students reported higher levels of engagement. |
| Dori & Belcher | 2005 | Higher education | USA | Q1, Q2 | TEAL classrooms. STEM Class sessions involved mini scattered lectures through the session with hand-on activities, supported with 13 tables with 9 students working in 3 members team | 350 | Students given questionnaires asking for most important elements that contributed to their understanding of the subject. Responses grouped into oral experiments, technology, written problems, and textbooks. | TEAL students improved their conceptual understanding of the subject matter to a significantly higher extent than their control group peer. |
| Ge , Yang, Liao & Wolfe | 2013 | Higher education | USA | Q2, Q3 | Five classes of 92 students (both undergraduates or graduates) and five professors from various disciplines (meteorology, biology, zoology, political science, and chemistry) + more four professors were interviewed. | 460 | Each participating class was examined through class observations, interviews, and surveys at the beginning and the end of the semester, asking students’ perceptions about: intrinsic motivation, problem-solving confidence, and problem-solving skills related to their subject domain. Descriptive statistics were conducted on the observation data, which were also qualitatively analyzed and coded. The interview data were coded, interpreted, categorized, and triangulated with the observation data to identify themes. | Students achieved significantly higher confidence scores in the posttest, but no significant differences in the measures of perceived intrinsic motivation problem-solving skills. Students’ self-efficacy and confidence in completing problem-solving tasks increased over time. Additionally, it was found that some professors used the ALC to its potentials while others used it minimally. |
| Gebre, Saroyan, & Aulls | 2015 | Higher education | Canada | Q3 | Students (n = 232) and professors (n = 13) using two new ALCs in large research university. | 232 | Multiple case study: Data from faculty collected using semi-structured interviews between weeks 3 and 10 of 13-week term, focusing on view of effective teaching in the context of the course, expected learning outcomes, instructional strategies, perceived role of computers in teaching and in realizing instructional goals, and type of applications used. | Interviews revealed three categories of effective teaching: Teacher centered, engagement centered, and learning/development centered activities. Faculty expressing second and third conceptions of effective teaching felt the affordances of the ALC were important, moreso than the technology in the room. 43% of students in classes whose profs had "transmission" view of effective teaching felt learning would be the same or better in a traditional classroom, compared with 27% and 8% of those in "engaging students" and "development" classes. |
| Harvey & Kenyon | 2013 | Higher education | USA | Q2, Q4 | Classrooms outfitted with five different seating types. | 863 | N = 863 students surveyed using Classroom Seating Rating Scale for Students with items on comfort and space, learning engagement, and interactivity. | Trapezoid tables and chairs on casters were more highly rated than the other three seating times on all survey items; no significant difference between these two seating types. |
| Henshaw, Edwards, & Bagley | 2011 | Higher education | USA | Q3, Q4 | Classroom outfitted with 48 fixed chairs swivel that 360 degrees. Seating placed into 4 groups of 12 students organized with two passages for movement. 10 instructors involved. | Not given | Surveys were distributed at the beginning of the semester to the teachers and one in the end for teachers and students + videos from 5 classes | Swivel chairs promote face to face interaction among students, facilitate instructor movement throughout the room, and minimize transition time between instructional modes. |
| Hyun, Ediger, & Lee | 2017 | Higher education | USA | Q2 | Sixteen university classes in various disciplines split between ALCs (5 classes) and traditional (11). | 16 classes | University of Minnesota STSS survey given once during weeks 8-9 of 10-week term, results grouped into satisfaction-group and satisfaction-individual. Correlative analysis conducted between several independent variables, with satisfaction variables dependent. | Active learning pedagogy and classroom type were significanty correlated with satisfaction-individual and satisfaction-group.Both active learning pedagogy and classroom type were also signficant predictors of both levels of student satisfaction. Student satisfaction (individual) was significantly increased in ALCs compared to measures of satisfaction in traditional classrooms. Student satisfaction was increased in ALCs vs traditional classrooms. |
| Imms & Byers | 2017 | Secondary education | Australia | Q2, Q4 | Church-related all-boys school; study involves 3 classes (n = 170) of year 7 students. Three learning spaces installed: Room 1 = traditional, Room 2 = altered to facilitate student-centered learning, Room 3 = polycentric with traditional and casual furniture plus digital and visual technology. Students put into groups "high ability" and "mixed ability" based on math scores. | 170 | Single-subject design: Two year 7 classes had main classrooms redesigned during first-term break — one is Room 2 and the other is Room 3 (third classroom kept as Room 1). Each class spent one term each in classroom during terms 2, 3, and 4. Students given Linking Teaching Pedagogy and Space (LTPS) survey every 3 weeks during these three terms. Summative test results in mathematics collated for comparison. | Perceptions of technology use improved in room 3 across all three classes. Statistically significant differences in ALC for perceptions of quality teaching for high- and one mixed-ability classes in room 3 and room 1 spaces; clear improvement for room 3 versus room 2 for high-ability students; mixed-ability class higher for for more 2 and 3 but difference was not statistically significant. High- and one mixed-ability classes showed significant improvement in "deeper levels of thinking" after room 3 intervention compared to room 1; remaining mixed-ability class showed significant improvement in room 2 space. Statistically significant improvement for student attitude following room 3 intervention; Statistically significant improvement for willingness to take on challenge following both room interventions across all three classes; Work beyond limit of expertise statistically significant only in room 3 for high-ability class. |
| Metzger | 2015 | Higher education | USA | Q2, Q3 | Students in two biology courses (sophomore level n = 144, upper level n = 48) were taught in ALCs using team teaching. Study attempts to examine the effects of team teaching in the ALC. | 192 | Survey: Students were given survey with one question "Having multiple instructors in the classroom at the same time helped me learn" rated on 5-point likert scale (1 = strongly agree); and one open-ended question “How did having multiple instructors in the classroom at the same time contribute to your learning?" (responses coded using grounded theory). | Mean responses to likert scale question were 1.82 and 1.53 for sophomore and upper level courses respectively. On open ended question, students indicated having two instructors helped in getting assistance on questions, helped students feel more supported, helped in delivering active learning activities, and helped in providing alternative viewpoints and examples. Difficulties of team teaching mentioned included distractions in class, lack of agreement on learning objectives and consistent messaging, and contradictory explanations of concepts. |
| Miller-Cochran, & Gierdowski | 2012 | Higher education | USA | Q2, Q4 | Sections of first-year writing course were conducted in a flexible-seat ALC in which students brought their own technology. Rooms used multiple LCD projectors, moveable whiteboards, and three kinds of moveable desks. | 195 | Survey: Students (n = 195) in sections holding class in the ALC were given a survey on preferences and perceptions relating to traditional spaces and ALCs. | 78% of students preferred flexible BYOT design over traditional fixed design; 5% preferred more fixed design, 17% no preference. 66% said the design of the ALC "somewhat contributes", "contributes", or "contributes highly" to their learning. |
| Muthyala & Wei | 2013 | Higher education | USA | Q1, Q4 | Two different forms of ALC introduced: "spoke" (7 tables radiating out from the center of room) and "node" (clusters of chairs on wheels). Introduced in "organic first" chemistry sequence in BS/Health Sciences program. Each class 10 minutes of minilecture followed by active learning activities. | 76 | Quasi-experimental: Exam scores and clicker question responses compared between the two sections. Exam measurement used "summative evaluations" that combined three exam grades, final exam, and final course grade. ANOVA and t-tests used to determine significance of differences between the two ALC sections. | No significant differences between the ALC layouts on either summative evaluation or on clicker questions. Time of the class meeting also made no significant difference. Non-significant but noticeable difference in Exam 1 averages between Spoke and Node layouts with Spoke being lower, but averages on later exams were higher for Spoke. |
| Nissim, Weissbluth, Scott-Weber & Amar | 2016 | Higher education | Israel | Q2, Q3, Q4 | Students in a teacher-training college, in courses held in ALCs designed as Steelcase LearnLabs with Node chairs and media:scape technological solutions. | 87 | Qualitative: n = 87 students in teacher-training program were administered the AL-POE questionnaire, once at beginning and once at the end of a semester. Questions from the AL-POE focused on improvement of creative ability, motivation to attend class, ability to achieve a higher grade, and overall engagement. | Survey responses showed statistically significant increases in both pedagogical practice and pedagogical solutions for overall engagement from the traditional space to ALC and in all individual factors for overall engagement. |
| Oliver-Hoy, Allen, Hunt, Hutson, & Pitts | 2004 | Higher education | USA | Q1 | Implementation of SCALE-UP (room holding 99 students) in introductory chemistry class using cAcL_2 pedagogical framework. Traditional class met twice weekly for 75 minutes with separate lab and problem solving sessions; SCALE-UP class met three times weekly for 100 minutes with no separate lab or problem sessions. | 99 | Quasi-experimental: Student performance on four exams during the semester recorded and compared between traditional and experimental sections. Three-way ANOVA used to look for interactions between exam scores, class, and major. | No significant different between individual test scores between two sections. However, exam*class interaction was significant; significant differences for experimental section on exams 2 and 4 but no positive significant differences on exams 1 and 3 The bottom 25% of student population in the experimental section outperformed the similar group in the traditional section on the last three exams. |
| Park & Choi | 2014 | Higher education | Korea | Q2, Q4 | 30-seat ALC was built for social science and literature classes, 5 circular tables with 6 students at each table, multiple projection screens, computer docking stations at each table. | 95 | Students in traditional classrooms were clustered into four parallel rows and given two surveys on satisfaction levels with the room. The results of this survey were combined into one collection of responses, called "Survey 1". Then students in the ALC were given a single survey on satisfaction ("Survey 2"). | Students in traditional room showed a preference for seating locations, creating a "golden zone" and "shadow zone". Different zones have effects on learning outcomes, with "golden zone" perceived as more beneficial for learning. Students found the ALC to be preferable to the traditional space in terms of sight lines, connections with classmates, having a sense of belonging to the class, fun, concentration, and attendance. |
| Pashak & Hagen | 2014 | Higher education | USA | Q2, Q3 | Undergraduate psychology course (n = 24) taught in a Steelcase LearnLab setup -- 4 tables, 6 students each plus PolyVision interactive whiteboards, personal whiteboards, and document camera. | 24 | Observational. Two research assistants observed class sessions and noted behaviors of both students (body movement, note-taking, etc.) and the professor (movement and pedagogical mode used). Also a survey given to students at the end of the course measuring affective responses. | Overall perceived level of student attention on 5-point likert scale was mean = 3.28, sd = 1.13. Timing of the class meeting had significant impact on attention. Significant one-way interaction between professor's behavior and teaching tool being used. Lower average of attention when the professor was lecturing or conducting question-answer, higher mean attention with less variation when engaged in Q&A plus supervision. Use of personal whiteboards was significantly related to higher levels of attention. In the survey, the use of swivel chairs, multiple screens, and interactive whiteboards had highest ratings; 37.5% of students had no significant dislikes regarding the class. |
| Rands & Gansemer-Topf | 2017 | Higher education | USA | Q2, Q3 | Four professors, nine students. Redesigned ALC for max 36 students. | 13 | Barkley’s (2010) classroom-based model of student engagement. 3 Focus groups where students were asked semi-structured questions regarding their interaction with others, with the physical and technological attributes of the classroom, and their perceptions of their own motivation and engagement. Data from the transcripts analyzed using a twocycle method of coding and analysis. | Respondents report that the classroom design creates a community of learners, affords movement and interaction, fosters greater connectedness, and affords greater ability to assess understanding and visualize thinking. |
| Salter, Thomason, Fox, & Lam | 2013 | Higher education | Hong Kong | Q2, Q3, Q4 | New building constructed with ALCs. Section of Intermediate Environmental Science course was studied -- traditional lecture followed by 2-hour problem-based learning session in the ALC. Section involved a new instructor with no ALC experience except for 3-day training workshop. | 14/not given | Case study: Class was observed and time spent on different types of learning activities recorded. Student learning measured through traditional exams and presentations on how they worked together to tackle the PBL problems — also weekly feedback using clickers. | In observations: 48% of time working collaboratively in small groups, 23% delivering or listening to student presentations, 16% listening to teacher address the entire class, 5% giving feedback with clickers. Students used technology in an integrated way. Students found large projection screens, small screens attached to tables, and laptops at the tables to be most important for learning. Students rated as important to the learning process the application of theory to real-life problems, listening to lectures given to the whole class, working in small groups, and presenting PBL work to the whole class. |
| Sawers, Wicks, & Mvududu | 2016 | Higher education | USA | Q3, Q4 | Thirty instructors, evenly split between males and females (48.3% each) | 30 | Demographic questionnaire and Classroom Utilization Survey administered along with open-ended questions. | Space has a greater impact on student engagement when used by faculty who have a more constructivist philosophy, therefore, focusing on building appreciation for a more constructivist teaching philosophy may be more effective. |
| Scott-Webber, Strickland, & Kapitula | 2013 | Higher education | USA | Q2, Q4 | 124 Students and educators, three different universities . Students were in Node Chairs class , LearnLab Media:scape class , and a combined Media:scape LearnLab classrom. | 124 | The AL-POE asked participants to compare their old/pre (row-by-column seating) environment with their new/post (SES’s intentionally designed: Node chair, Verb table, Space) through an online tool Qualtrics, 2 emails one 6-8 after the begining of the semster and on in the end. The AL-POE is structured in four sections:(1) demographics and baseline information, (2) learning practices, (3) solutions, and (4) perception of outcomes. Construct validity was assessed through exploratory factor analysis (EFA) and individual item analysis on the polychoric correlation matrix | Statistically signficant differences between ALC and traditional spaces (with ALC more favorably rated) on all 12 of the dimensions of engagement measured by the AL-POE. |
| Stover & Zisweiler | 2017 | Higher education | USA | Q2, Q4 | Study involved n = 417 students enrolled in classes of greater than 70 students at a university. Classes in Spring 2015 semester taught in a traditional auditorium with fixed seating and instructor lecturing from front podium; classes in Fall 2015 taught using active learning in an ALC by three faculty trained to use ALCs -- one cutting lecture by half and replacing with active learning, another going from 85% lecture to 80%, a third incorporating a flipped learning approach. | 417 | Quantitative: Survey given to students in the final week of semester consisting of 34 self-report items from the Community of Inquiry survey. | ALC had a statistically significant negative effect on perceived instructor presence in evening section of teacher 2 and with teacher 3. ALC had statistically significantly positive impact on social presence - interaction in Class 1. Statistically significant negative impact on social presence - participation in Class 3. No statistically significant impact in any class on cognitive presence. |
| Taylor | 2009 | Higher education | USA | Q2 | Two science classes taught in the same semester in a studio space (one class with n = 25 students and the other with n = 9). | 34 | Four student surveys administered at equal intervals during the semester with items on general impressions and specific uses and features of the studio. | Students commonly report positively on physical comfort of the space and rated the ALC as a more forgiving environment for communication, movement, and engagement. |
| Van Horne, Murniati, Saichaie, Jesse, Florman, & Ingram | 2014 | Higher education | USA | Q2, Q3, Q4 | ALCs constructed across campus --- large rooms, 9-person round tables, projectors and wall-mounted monitors, glass whiteboards. | Varies | Qualitative: Semi-structured interviews with instructors at beginning and end of one semester on how they adapted their teaching for the ALCs and focus groups with students, and their attitudes on teaching in ALC; also student focus groups on helpful/nonhelpful aspects of learning in ALC. | Instructors reported that ALCs enabled them to use teaching methods that could not be supported in regular classrooms. Instructors redesigned activities in ALCs using the tools in the classrooms. Activities in the classrooms work well when there is a mechanism to make sure that students prepared the materials prior to coming to class and stay on tasks during the activities. Instructors reported that students benefit from TILE environment when they are given authority for sharing their work. Student focus group results were not reported. |
| Walker, Brooks, & Baepler | 2013 | Higher education | USA | Q1, Q2, Q3 | Introductory biology course for non-biology majors, one instructor and two sections, one section in an ALC and the other in a traditional space. (n = 161 and 102 respectively) | 263 | Quasi-experiment. Surveys on their perceptions of their spaces given during last week of class; personal data collected from students; observations done on randomly selected 50% of students and recorded levels at which students were on task, as well as specific learning behaviors being exhibited; final grades in each section recorded. | Students in ALC performed significantly better on course grades (half letter grade higher) than their initial ACT scores predicted. ALC instructors spent more time consulting and leading group activities and less time at podium than instructors in traditional space. Significant positive correlations between the use of ALCs and group activities. ALC students reported higher levels of engagement. |
| Whiteside, Brooks, & Walker | 2010 | Higher education | USA | Q1, Q2 | Introductory biology course was taught in new ALCs with round tables seating 9 students each, with switchable laptop technology to feed student screens to large projectors, along with glass whiteboards around the room. | 190 | Quasi-experimental: Pre-post design with one section in ALC and the other in a traditional classroom. Only significant variable was space and ACT score with the traditional section having a higher ACT average. Instructional space was varied, and course grades measured as output. Also observational: Classroom observation protocol was developed and used to observe activities in both sections. | See Brooks (2011) for quantitative results on predicted ACT scores. Qualitatively: Instructor in ALC conducted more discussion and moved through the space more regularly than in a traditional space. Students reported significantly higher ratings for ALCs on contribution to their class engagement, enrichment of their experiences, flexibility of learning, and fit of classroom to the course. Significant differences in how students evaluate the rooms based on rural vs. city background of students and junior/senior vs. freshman/sophomore classification. |
| Whiteside, Jorn, Duin, & Fitzgerald | 2009 | Higher education | USA | Q2, Q3 | Two classrooms (one for engineering, another for biological sciences) renovated into ALCs: round tables with 9 seats at each, whiteboards, multiple projectors in the room. | 168/13 | Qualitative: Surveys and exit interviews conducted with faculty and students using the room throughout 2007-2008 academic year (n = 17 instructors, n = 168 students) along with class observations (n = 29), all focused on faculty and student attitudes and perceptions. | Instructors reported a shift in perceived role (from teacher to "coach") with deepened student/professor relationships. Student perceptions overall favorable with ALC's perceived as effective for team/collaborative projects, feeling more connected, and having discussion encouraged. Both students and faculty reported technology issues with user interfaces and access issues. More than 85% of students recommend the ALCs. |
